# Integrating genome‐ and transcriptome‐wide association studies to uncover the host–microbiome interactions in bovine rumen methanogenesis

**DOI:** 10.1002/imt2.234

**Published:** 2024-09-03

**Authors:** Wei Wang, Zhenyu Wei, Zhuohui Li, Jianrong Ren, Yanliang Song, Jingyi Xu, Anguo Liu, Xinmei Li, Manman Li, Huimei Fan, Liangliang Jin, Zhannur Niyazbekova, Wen Wang, Yuanpeng Gao, Yu Jiang, Junhu Yao, Fuyong Li, Shengru Wu, Yu Wang

**Affiliations:** ^1^ Department of Animal Genetics Breeding and Reproduction, College of Animal Science and Technology Northwest A&F University Yangling China; ^2^ Department of Animal Nutrition and Environmental Health College of Animal Science and Technology Northwest A&F University Yangling China; ^3^ Department of Clinical Veterinary College of Veterinary Medicine Northwest A&F University Yangling China; ^4^ School of Ecology and Environment Faculty of Life Sciences and Medicine Northwestern Polytechnical University Xi'an China; ^5^ Key Laboratory of Livestock Biology Northwest A&F University Yangling China; ^6^ Department of Animal Science and Technology College of Animal Sciences Zhejiang University Hangzhou China

**Keywords:** GWAS, Holstein cattle, host genetics, methanogenesis, rumen microbiota, TWAS

## Abstract

The ruminal microbiota generates biogenic methane in ruminants. However, the role of host genetics in modifying ruminal microbiota‐mediated methane emissions remains mysterious, which has severely hindered the emission control of this notorious greenhouse gas. Here, we uncover the host genetic basis of rumen microorganisms by genome‐ and transcriptome‐wide association studies with matched genome, rumen transcriptome, and microbiome data from a cohort of 574 Holstein cattle. Heritability estimation revealed that approximately 70% of microbial taxa had significant heritability, but only 43 genetic variants with significant association with 22 microbial taxa were identified through a genome‐wide association study (GWAS). In contrast, the transcriptome‐wide association study (TWAS) of rumen microbiota detected 28,260 significant gene–microbe associations, involving 210 taxa and 4652 unique genes. On average, host genetic factors explained approximately 28% of the microbial abundance variance, while rumen gene expression explained 43%. In addition, we highlighted that TWAS exhibits a strong advantage in detecting gene expression and phenotypic trait associations in direct effector organs. For methanogenic archaea, only one significant signal was detected by GWAS, whereas the TWAS obtained 1703 significant associated host genes. By combining multiple correlation analyses based on these host TWAS genes, rumen microbiota, and volatile fatty acids, we observed that substrate hydrogen metabolism is an essential factor linking host–microbe interactions in methanogenesis. Overall, these findings provide valuable guidelines for mitigating methane emissions through genetic regulation and microbial management strategies in ruminants.

## Highlights

- Using large‐scale matched samples, we reveal the impact of host genetics variants and rumen gene expressions on rumen microbiota in Holstein cattle.
- Host genetic factors average explained approximately 28% of the microbial abundance variance, whereas rumen gene expression explained 43%.
- The transcriptome‐wide association study is a promising method for exploring the associations between gene expression and complex phenotypes in direct effector organs.
- Host–microbial interactions in bovine rumen methanogenesis are possibly involved in substrate hydrogen metabolism and transport.

## Graphical abstract

We systematically evaluated the relationship between host and microorganisms using matched host genome, rumen transcriptome, and microbiome data from a cohort of 574 Holstein cattle. The associations between rumen gene expression and rumen microbiota abundance via transcriptome‐wide association study (TWAS) may provide more molecular markers at the gene level, which bridge the host–microbe interactions. Methanogens belonging to *Methanobrevibacter* genus are common hydrogenotrophic methanogenic archaea, which usually utilize hydrogen (H_2_) and carbon dioxide (CO_2_) produced from microbial fermentation as substrates to produce methane (CH_4_). By combining multiple correlation analyses based on host TWAS genes, rumen microbiota, and volatile fatty acids, we illustrated that host–microbe interactions in the hydrogenotrophic methanogenesis pathway are possibly involved in substrate hydrogen metabolism and transport. This study broadens our insights into the potential mechanisms of host–microbe interactions in rumen methanogenesis.

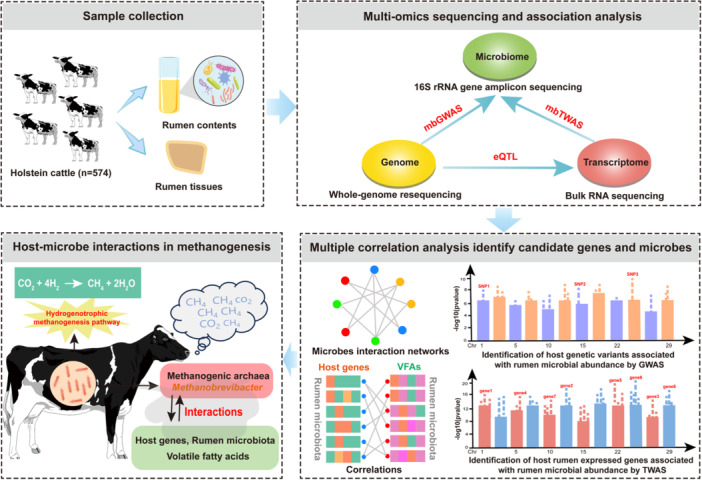

## INTRODUCTION

Methane is one of the six greenhouse gases that is second only to carbon dioxide in its performance for global warming [1]. Mitigating methane emissions from livestock production is crucial to achieve carbon neutrality in China [2]. The rumen microbiota of ruminants is responsible for the production of methane and contributes about 18% of its total anthropogenic emissions [3]. Cattle, as crucial domestic ruminant, contribute the majority of livestock production emissions of methane [4, 5], which is attributed to their strong fermentation function of rumen microorganisms [6]. Methane production is closely related to the abundance of methanogenic archaea in the rumen, which are mainly from the *Methanobrevibacter* genus [7]. The abundances of methanogens belonging to the *Methanobrevibacter* genus (e.g., *M. gottschalkii*, *M. smithii*, *M. boviskoreani*, *M. millerae*, and *M. thaurei*) were positively correlated with methane emissions [8, 9, 10, 11, 12, 13], which are common hydrogenotrophic methanogenic archaea usually utilizes hydrogen (H_2_) and carbon dioxide (CO_2_) produced from microbial fermentation as substrates to produce methane (CH_4_) [10, 14]. In addition to its negative environmental impact, enteric methane emissions result in a 2%–12% loss of gross energy intake for the host [15, 16]. Therefore, it has extremely been desirable to find methods to modulate the rumen microbiome to reduce methane emissions of ruminants all the time.

The gut microbiota is shaped by diet, host, environment, and other factors. The regulation of the gut microbiota by host‐derived molecules provides a new perspective for understanding host–microbe interactions [17]. An increasing number of studies have highlighted the important role of host genetics on gut microbiota in human and animals [18, 19]. Heritability estimation of gut microbiota helps to understand the proportion of host genetic factors that explain changes in microbial abundance. According to TwinsUK population studies, 5.3%–8.8% of bacterial taxa have heritability estimates greater than 0.2 in stool samples [20, 21]. The host genetic factors of ruminants seem to have a greater impact on the rumen microbiota. According to previous studies, approximately 34% of microbial taxa and 64% core genera had significant heritability from a cohort of 709 beef cattle and 1150 male sheep lambs [22, 23]. In addition, the rumen *Methanobrevibacter* genus of dairy cows also has been reported to have moderate heritability (*h* ^2^ = 0.22) [24]. In recent years, numerous studies have focused on identifying the associations between host genetics variants and microbial abundance variation via genome‐wide association study (GWAS) using the microbiome as complex traits [25, 26, 27, 28]. Using GWAS, researchers have identified many single nucleotide polymorphisms (SNPs) in cattle, which are related to microbiota composition, feed efficiency, host immunity, and metabolism [22, 29, 30]. However, although methane emissions and methanogen abundance have been reported to have moderate heritability, no major loci have been identified in multiple studies through GWAS [22, 29, 31]. Compared to GWAS, transcriptome‐wide association studies (TWAS) have been developed to interpret the relationship between gene expression and phenotype, which is of great value for explaining the genetic basis of complex phenotypes as well as providing gene‐level associations and have been conducted across various traits and tissues [32]. Interestingly, establishing the correlations between host genes and rumen methanogens via the TWAS approach is a promising strategy for host regulation of methane emission. Notably, in most studies, TWAS tests usually involve genetically predicted expression using summary data rather than dynamic correlations between gene expression of the effector organs and phenotypes from paired samples, which may cause false hits and bias [33]. At present, a number of studies have integrated GWAS and TWAS using paired samples to identify molecular markers related to agronomic traits in plants [34, 35, 36]. However, to the best of our knowledge, the integrated GWAS and TWAS analyses of large‐scale paired samples have not been used in animal studies thus far, especially in gastrointestinal microorganisms.

**Figure 1:**
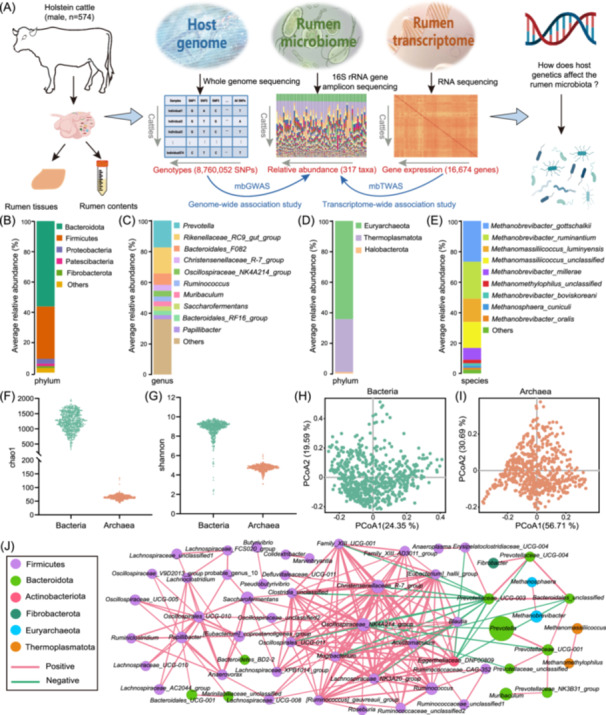
Study design and the composition and community structure of rumen microbiota. (A) Workflow of the integrated rumen genome, transcriptome and microbiome to uncover the host genetic basis of rumen microbiota. The composition and abundance of bacterial taxa at the phylum (B) and genus (C) levels. The composition and abundance of archaeal taxa at the phylum (D) and species (E) levels. (F, G) The ⍺ diversity was determined using chao1 and Shannon indices. (H, I) The β diversity principal coordinates analysis plot based on Bray–Curtis distances for bacteria and archaea at genus level. (J) Interaction networks of taxa at the genus level. Only correlation coefficients <− 0.5 or > 0.5 and adjusted *p* values <0.05 are displayed. The node size represents the average relative abundance. mbGWAS, genome‐wide association study of microbiota; mbTWAS, transcriptome‐wide association study of microbiota; rRNA, ribosomal RNA; SNP, single nucleotide polymorphism.

In this study, we aim to bridge the host and microbiome in rumen methanogenesis. We hypothesized that the relationship between rumen genes and rumen microbes is more direct than host genetic factors, and the host influences microbial changes by driving rumen gene expression, thereby regulating methane emissions. To address these hypotheses, we conducted matched genome, transcriptome, and microbiome sequencing of the rumen through a single large‐scale cohort of 574 Holstein cattle and performed genome‐wide association study of microbiota (mbGWAS) and transcriptome‐wide association study of microbiota (mbTWAS) to identify the genetic variants and rumen genes influencing the rumen microbiota (Figure 1A). These works will explore the possibility of genetically regulating rumen microbiota to mitigate methane emissions in cattle and broaden our insights into the potential mechanisms of host–microbe interactions in rumen methanogenesis.

## RESULTS

### Landscape of rumen microbiota using a single large cohort of Holstein cattle

We performed 16S ribosomal RNA (rRNA) gene amplicon sequencing of rumen content samples from 574 cattle. After quality filtered, a total of 32,622,851 high‐quality sequences were obtained from both bacteria and archaea. An average of 35,336 (ranging from 14,117 to 60,769) and 22,234 (ranging from 8795 to 33,240) tags per sample for bacteria and archaea, respectively (Figure S1A,D). The reads with 99% similarity were clustered into amplicon sequence variants (ASVs). We obtained a total of 5974 and 3588 ASVs for bacteria and archaea (Tables S1 and S2), with an average of 1175 (ranging from 317 to 1833) and 65 (ranging from 43 to 135) ASVs per sample for bacteria and archaea, respectively (Figure S1B,E). After taxonomic annotation, 269 taxa at five levels (including 13 phyla, 19 classes, 33 orders, 60 families, and 144 genera) for bacteria and 48 taxa at six levels (including three phyla, four classes, four orders, four families, eight genera, and 25 species) for archaea with a prevalence >20% were detected (Figure S1C,F and Table S3). The dominant bacterial phyla were Bacteroidota (56.4%), Firmicutes (34.5%), and Proteobacteria (3.3%) (Figure 1B). The dominant bacterial genera were *Prevotella* (17.7%), *Rikenellaceae_RC9_gut_group* (16.9%), and *Bacteroidales_F082* (7.6%) (Figure 1C). The dominant archaeal phyla were Euryarchaeota (64.4%) and Thermoplasmatota (34.7%) (Figure 1D). The dominant archaeal species were *M. gottschalkii* (26.8%), *M. ruminantium* (24.2%), and *Methanomassiliicoccus luminyensis* (15.3%) (Figure 1E). Microbial community structure and diversity were analyzed by ⍺ and β diversity with different indices, and the results showed that the microbial richness of bacteria was greater than that of archaea (Figure 1F,G) and the tested individuals had a similar microbial composition and community structure (Figure 1H,I). To explore the interactions of rumen microbial taxa, a correlation network at the genus level was analyzed using a Spearman rank correlation coefficient, and we identified 58 strongly correlated taxa (|*r*| > 0.5, adjusted *p* < 0.05) (Figure 1J). Among them, a number of genera with high connectivity and presence in all individuals, such as *Oscillospiraceae_NK4A214_group*, *Christensenellaceae_R‐7_group*, *Lachnospiraceae_NK3A20_group*, *Ruminococcus*, and *Prevotellaceae_UCG‐003*, represent a core rumen microbiota of Holstein cattle. We also predicted the functional pathways of the 16S rRNA gene from all ASVs using PICRUSt2. The correlation between taxa and functional pathways showed that *Prevotella* is associated with l‐rhamnose degradation. The *Christensenellaceae_R‐7_group* is associated with the superpathway of (R, R)‐butanediol biosynthesis and myo‐, chiro‐, and scillo‐inositol degradation. *Methanobrevibacter* was positively associated with many pathways, such as coenzyme M biosynthesis, factor 420 biosynthesis, and methanogenesis from H_2_ and CO_2_, which are the main functional pathways of methane production (Figure S2).

### Host genetic factors affect the rumen microbiota

**Figure 2:**
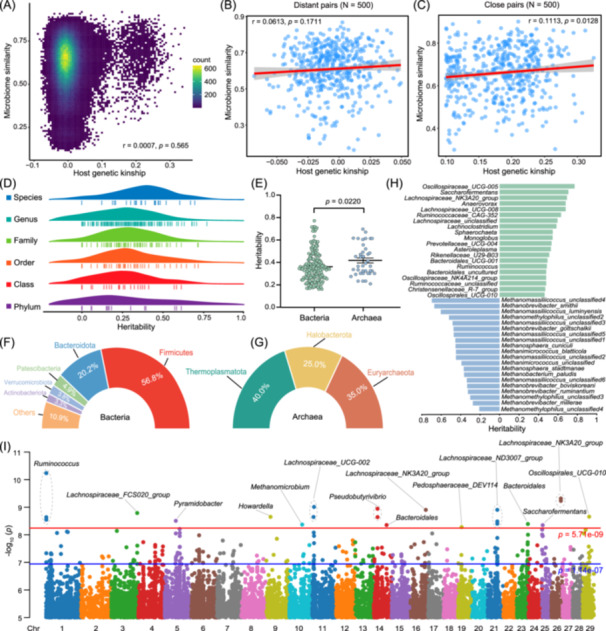
Study on the relationship between host genetics and rumen microbiota. (A) The relationship between host genetic kinship and Bray–Curtis similarity in all pairs. We randomly selected 500 genetically more distant (B) and close (C) pairs of individuals to calculate correlations. (D) Heritability estimation of 317 single taxa traits. (E) Comparison of heritability between bacterial and archaeal taxa. (F, G) The percentage of heritable taxa at different phylum levels for bacteria and archaea. (H) The top 20 heritable taxa in bacteria and archaea. (I) Genome‐wide association of cattle genetic variants and heritable rumen microbial variations. Each single nucleotide polymorphism (SNP) was tested against each of the 223 heritable taxa and the Manhattan plot shows the lowest resulting *p* value for each SNP. The red line indicates the genome‐wide significance level (*p* < 5.71 × 10^−9^).

Individual genetic information of 574 Holstein cattle yielded a total of 10.5 Tb of raw data using whole‐genome sequencing, with an average coverage of sevenfold per individual. After strict quality control, the final set of 8,760,052 SNPs was obtained. Principal component analysis of these SNPs was used to assess population structure (Figure S3A). Genome‐wide genetic variants distribution on 29 autosomes (Figure S3B) and gene annotation revealed that the majority of SNPs were present in either intergenic regions (56.3%) or intronic regions (35.9%) (Figure S3C and Table S4). To investigate whether host genetics impact the rumen microbiota, a correlation analysis was performed between host genetic kinship and Bray–Curtis similarity using the Mantel test. In view of the fact that most pairs of individuals showed a low degree of genetic relatedness (Figure 2A), we randomly selected 500 genetically more distant and close pairs of individuals to calculate the correlations, and the correlation coefficients were 0.06 and 0.11, respectively (Figure 2B,C). These results suggested that weakly positive correlations exist between host genetic kinship and microbiota similarity. We then evaluated the heritability (*h* ^*2*^) of 317 microbial features, including 171 abundance and 146 presence/absence traits (Figure 2D and Table S5). We found that host genetics factors average explained approximately 28% of the microbiome variance, and approximately 70% (223/317) of taxa had significant heritability (likelihood ratio test, *p* < 0.05). The average heritability of archaea (0.41 ± 0.13) was higher than bacteria (0.36 ± 0.14) (Figure 2E). Among these heritable bacterial taxa more than 50% belong to Firmicutes (Figure 2F), and the heritable archaea were mainly in Thermoplasmatota and Euryarchaeota (Figure 2G), such as *Oscillospiraceae_UCG‐005* (*h* ^*2*^ = 0.77), *Christensenellaceae_R‐7_group* (*h* ^*2*^ = 0.46), and *M. gottschalkii* (*h* ^*2*^ = 0.47) (Figure 2H). In short, host genetics affects the rumen microbiota to some extent, and the heritability of the rumen core microbiota may be nearly universal.

To gain insights into the host additive genetic effects on rumen microbiota phenotypes, we performed a genome‐wide association study (mbGWAS). We investigated the association between 8,760,052 autosomal genetic variants and 223 heritable taxa from 574 cattle. There was no evidence for test statistic inflation in the GWAS (median genomic inflation factor (*λ*_GC_) = 1.009) (Figure S4). We identified 43 SNP‐taxon associations at a genome‐wide *p* value threshold of 5.71 × 10^−9^, which involved 22 taxa traits and 17 independent loci (Figure 2I and Table S6). For instance, the significant signal loci at 42.9 Mb region on chr21 were associated with *Lachnospiraceae_ND3007_group* abundance, where the most significant SNP (21:42903221, *p* = 1.25 × 10^−9^) was located in an intron of the *AKAP6* gene (Figure S5A–D). Moreover, in our study, we obtained RNA‐seq data from rumen tissues, and these large‐scale paired genome and transcriptome samples provide an unprecedented opportunity to detect *cis*‐eQTLs in rumen. In total, 8,760,052 SNPs and 16,674 genes in 454 individuals were performed *cis*‐eQTL mapping using a linear regression model in FastQTL software. We detected 499,502 significant variant‐gene pairs, including 1619 unique eGenes and 416,109 unique eSNPs. To confirm whether host genetic variation affects rumen microbes by affecting rumen gene expression, we conducted a colocalization analysis of *cis*‐eQTLs and GWAS loci using COLOC and found 86 possibly colocalized loci from 770 common SNPs (Table S7). For instance, we found that a *cis*‐eQTL of the *POLM* gene in the rumen was colocalized with the mbGWAS locus of *Lachnospiraceae_FCS020_group* abundance trait (Figure S6A–D). Taken together, these results suggest that host genetics could influence and regulate rumen microorganisms. However, the mechanisms underlying host genetics and taxon abundance associations, as well as direct effector organs, are unclear. Hence, it is, particularly, vital and urgent to establish the interactions between rumen effector organ gene expression and rumen microbiota.

### TWAS atlas of rumen microbiota

**Figure 3:**
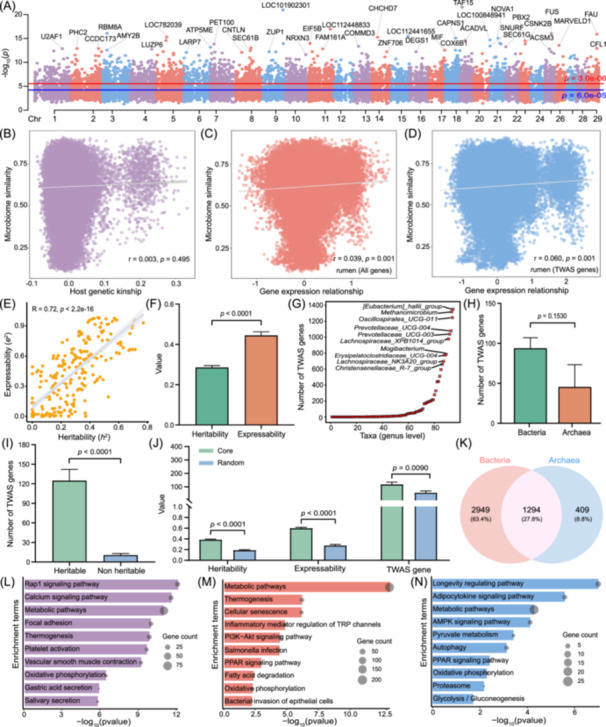
Characteristics of the transcriptome‐wide association study (TWAS) atlas of rumen microorganisms. (A) Transcriptome‐wide association of rumen gene expression and rumen microbial abundance. The Manhattan plot shows the lowest resulting *p* value for each gene. (B) The relationship between host genetic kinship and microbiome similarity in 454 paired individuals. The correlation between gene expression relatedness and microbiome similarity, which was constructed in all genes (C) and TWAS genes (D). (E) Spearman's correlation of heritability and expressability. (F) Comparison of the difference between heritability and expressability. (G) The number of TWAS genes of taxa at genus level. (H) Comparison of the number of TWAS genes in each taxon between bacteria and archaea. (I) Comparison of the number of TWAS genes between heritable taxa (*h* ^2^ > 0.2) and nonheritable taxa (*h* ^2^ ≤ 0.2). (J) The differences in heritability, expressability, and TWAS genes among the rumen core taxa (prevalence > 80%) and random taxa (prevalence 20%–80%). (K) The overlapping TWAS genes between rumen bacteria and archaea. (L‐N) Functional enrichment analyses for bacteria and archaea shared TWAS genes, bacteria‐specific genes, and archaea‐specific genes, respectively. For the group comparison, the values are the means ± SEMs. A *p* value less than 0.05 was considered significant for all tests.

Given that most GWAS loci are often difficult to interpret, a large amount of effort has been devoted to identify the trait‐associated genes and understanding the genetic architecture of complex traits by implementing TWAS with predicted gene expressions from GTEx summary data in the last few years [32, 37, 38]. In our current study, we collected rumen samples with dynamically corresponding gene expression and microbial abundance data. Therefore, for the first time, we constructed a TWAS atlas of rumen microbiota based on the association between 16,674 genes and 317 taxa. We detected 28,260 significant gene–microbe associations (*p* < 3 × 10^−6^), which include 16,423 positive correlations and 11,837 negative correlations, and included 210 taxa and 4652 unique genes (Figure 3A and Figure S7). We found that more gene biomarkers were found using TWAS than using GWAS and *cis*‐eQTL, and only 53 shared genes were obtained by three methods (Figure S8). Therefore, we hypothesized that the dynamic changes in rumen gene expression and microbial abundance have a more direct relationship. To further confirm this, we evaluated the correlation between the gene expression relationship matrix (ERM) and microbiome similarity matrix. The results displayed that the correlation between gene expression and microbiome similarity was stronger (*r* = 0.039, *p* = 0.001) than that of host genetics (*r* = 0.003, *p* = 0.495), and this effect was more pronounced for TWAS genes than for all genes (*r* = 0.060, *p* = 0.001) (Figure 3B–D). We then evaluated the “expressability” of all taxa traits based on methods similar to heritability and found that the rumen gene expression average explained approximately 43% of the variance in the microbiome (Table S8). Heritability and expressability were significantly positively correlated (*r* = 0.72, *p* < 2.2e−16) (Figure 3E), and the expressability significantly higher than heritability (Figure 3F). Moreover, the TWAS correlation features showed that the *[Eubacterium] hallii_group* and *Methanomicrobium* with the most TWAS genes for bacteria and archaea, respectively (Figure 3G). The number of TWAS genes was not a significant difference between bacterial and archaeal taxa (Figure 3H). The heritable rumen microbiota (*h* ^*2*^ > 0.2) had more TWAS genes (Figure 3I). Compared with random microbiota, rumen core microbiota (prevalence >80%) had higher heritability, expressability, and more TWAS genes (Figure 3J). To clarify the function of rumen microorganisms TWAS genes, we compared the differences in functional enrichment of TWAS genes between bacteria and archaea. We found that the bacteria and archaea shared 1294 genes (Figure 3K), these genes were significantly enriched in Rap1 signaling pathway, calcium signaling pathway, and metabolic pathways (Figure 3L). The bacterial‐specific genes were significantly enriched in metabolism, immunity, and pathogenic microbial infection pathways (Figure 3M). The archaeal‐specific genes were mainly enriched in longevity regulating pathway, pyruvate metabolism, and glycolysis/gluconeogenesis (Figure 3N). In addition, we are the first to conduct TWAS analysis with a relatively large sample size for a single tissue. Therefore, we evaluated the effect of different sample sizes (100, 200, 300, 400, and all 454) on TWAS analysis, and the results showed that with increasing sample size, the number of TWAS genes gradually increased. For example, when 100 samples were used, *Christensenellaceae_R‐7_group* had only two significantly associated genes, whereas when the sample size was increased to 200, there were 49 significantly associated genes. The number of taxa with significantly associated genes gradually stabilized after 200 samples (Figure S9A–C). Based on this, we suggest that when performing TWAS analysis, the sample size should be larger than 200 to obtain ideal results.

### Integrating GWAS and TWAS reveals host‐associated features of rumen archaea

**Figure 4:**
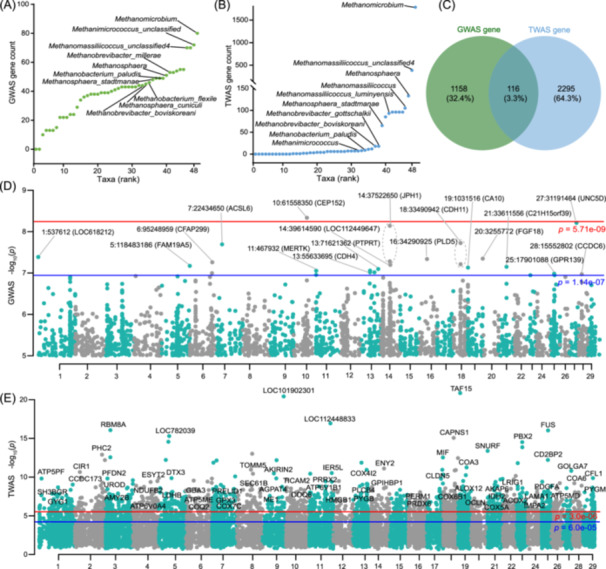
The regulation effects of the host on rumen archaea. (A) The number of genome‐wide association study (GWAS) nearest genes of 48 rumen archaeal taxa at *p* < 1 × 10^−5^ threshold. (B) The number of transcriptome‐wide association study (TWAS) genes of 48 rumen archaeal taxa at *p* < 1 × 10^−5^ threshold. (C) The shared genes captured by GWAS and TWAS approaches. (D) The GWAS results of rumen archaea. (E) The TWAS results of rumen archaea. The Manhattan plot shows the lowest resulting *p* value for each SNP and gene. The red and blue lines represent genome‐wide significance and suggestive significance thresholds, respectively.

To further investigate the associations among host genetic variants and rumen gene expressions with rumen archaeal abundance, we conducted GWAS and TWAS for 48 archaeal taxa traits. According to the significant threshold of *p* < 1 × 10^−5^, we identified 3049 SNPs using GWAS, and these SNPs annotated 1274 nearest genes (Figure 4A). At the same time, we obtained 3201 significant gene‐taxa associations using TWAS, covering 2411 unique genes and 37 taxa traits (Figure 4B). By comparing these two gene sets, we found that only 3.3% of shared genes were captured by both GWAS and TWAS approaches (Figure 4C). Remarkably, at the Bonferroni‐corrected *p* values threshold, only one significant SNP locus (*p* < 5.71 × 10^−9^) was associated with *Methanomicrobium* abundance according to GWAS (Figure 4D). In contrast, a total of 1703 significant associated genes (*p* < 3 × 10^−6^) were identified by the TWAS method (Figure 4E). These results suggested that for some complex phenotypes, such as microbiome abundance, regulated by host microeffect polygenes, only on the GWAS method may not be able to find effective genetic markers. On the contrary, examining the correlations between gene expression and phenotype via TWAS may provide additional molecular markers at the gene level and may become an important tool for revealing the molecular mechanism of complex phenotypes. Therefore, these rumen archaea‐associated host genes provide a new opportunity to resolve methane emissions from the perspective of host–microbe interactions.

### Multiple correlation analysis discovers promising genes and microbes in methanogenesis

**Figure 5:**
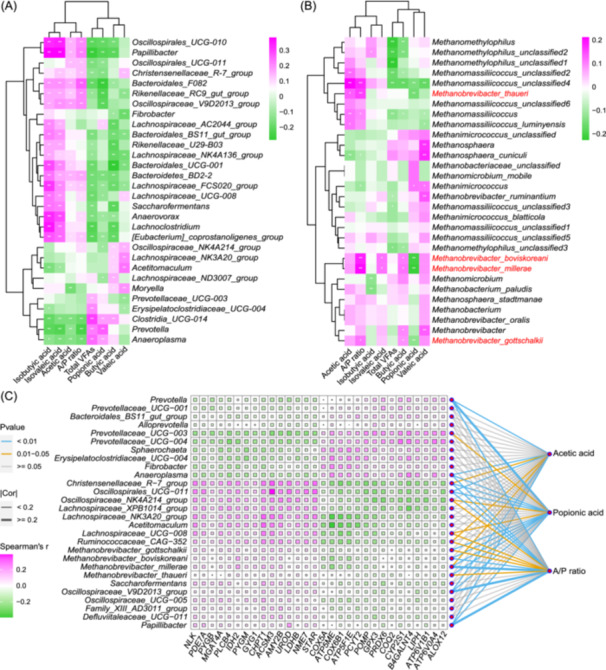
Multiple correlation networks of genes–taxa–volatile fatty acids (VFAs). (A) The correlations between rumen bacterial taxa and VFAs. (B) The correlations between rumen archaeal taxa and VFAs. (C) Multiple correlation analyses among host rumen genes, rumen microbiota, and volatile fatty acids. Only correlation coefficients <− 0.2 or >0.2 and adjusted *p* values < 0.05 are displayed. *p < 0.05, **p < 0.01.

To uncover the role of host rumen epithelial genes, rumen microbiota, and microbial volatile fatty acids (VFAs) in methanogenesis, we integrated a triple interaction relationship network (genes‐taxa‐VFAs). First, considering that VFAs could indirectly reflect rumen fermentation patterns and methane emission levels, we measured rumen VFA concentration (Table S9), and the correlation analysis was performed between the taxa and VFAs for bacteria and archaea, respectively. The results showed that the *Bacteroidales_F082* was positively correlated with acetic acid and A/P, the *Prevotella* was positively correlated with popionic acid, and the *Clostridia_UCG‐014* was positively related with total VFAs (Figure 5A). Notably, for archaea, we found that the four species belong to *Methanobrevibacter* genus (e.g., *M. boviskoreani*, *M. millerae*, *M. thaueri*, and *M. gottschalkii*) were positively correlated with acetic acid and A/P but negatively correlated with propionic acid (Figure 5B). Subsequently, based on these four methanogens, we constructed a microbial interaction network and found 36 rumen bacteria (|*r*| > 0.2) associated with them (Figure S10). To explore host rumen expression genes related to the regulation of methanogenesis, we combined all the TWAS‐associated genes of the above 40 taxa and selected 252 genes enriched in metabolic pathways (Figure S11). We next performed the correlation analysis between these host rumen genes and rumen microbiota. Based on these results, we established a comprehensive relationship network among host rumen genes, rumen microbiota, and rumen VFAs with methanogenic archaea (Figure 5C and Tables S10 and S11).

**Figure 6:**
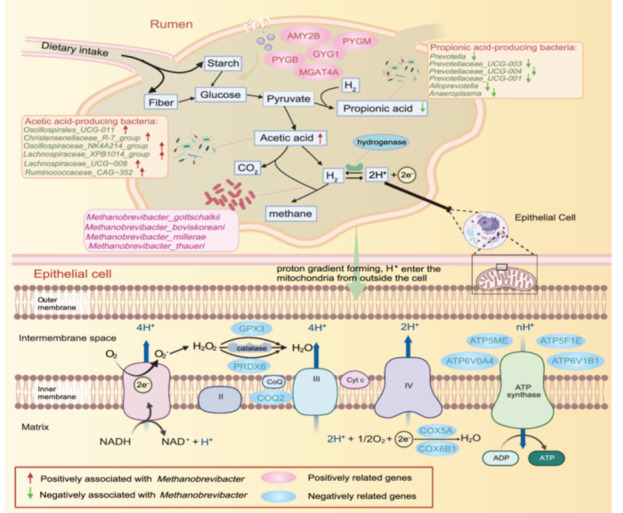
Overview of putative ruminal carbohydrate and hydrogen metabolism pathways in methanogenesis. The upper part illustrates the potential process by which high expression of the rumen epithelial amylase and glycogen genes leads to more extensive breakdown of starch into glucose in the rumen, which reduces the niche advantage of starch‐utilizing bacteria and increases opportunities for acetate‐producing microorganisms to acquire nutrients. This promotes a shift in rumen fermentation towards acetate‐type fermentation. Acetate‐type fermentation results in increased hydrogen production, which in turn enhances the abundance of *Methanobrevibacter*, a genus of methanogenic archaea. The lower part exhibits the potential process of electron transfer and hydrogen ion transport in the mitochondrial oxidative respiratory chain of host rumen epithelial cells. When genes for rumen epithelial hydrogen ion transport and oxidative phosphorylation are highly expressed, the pathways for hydrogen utilization in the rumen increase, thereby reducing the abundance of *Methanobrevibacter* in the rumen. Candidate microbes and host genes are highlighted by arrows and ovals of different colors.

Methane production is considered the main hydrogen (H_2_) sink in the rumen, which is synthesized by methanogens with H_2_ and CO_2_ as substrates, therefore, hydrogen metabolism and its associated microbes in the rumen may be closely related to low‐ and high‐methane phenotypes [39, 40]. From our above multiple correlation analyses, we found that some taxa (e.g., *Oscillospirales_UCG‐011*, *Christensenellaceae_R‐7_group*, and *Oscillospiraceae_NK4A214_group*) were positively correlated with four methanogens (including *M. boviskoreani*, *M. millerae*, *M. thaueri*, and *M. gottschalkii*), acetic acid, and A/P ratio, which may be potential acetic acid‐producing bacteria (Figures 5A and 6 and Figure S10). These microbes fermented to produce acetic acid and a large amount of hydrogen, and this hydrogen could be used by methanogens archaea to produce methane. Several other taxa (e.g., *Prevotella*, *Prevotellaceae_UCG‐003*, and *Anaeroplasma*) were negatively correlated with four methanogens but positively correlated with propionic acid, implying that these microbes may be potential propionic acid‐producing bacteria (Figures 5A and 6 and Figure S10). The propionic acid production could competitively consume H_2_ and reduce H_2_ availability for methanogenesis [31, 41]. This is consistent with a study in buffalos in which high *Prevotella* abundance was associated with low methane emissions [42]. At the host gene level, we observed that several host rumen genes enriched in starch and sucrose metabolism pathway (e.g., amylase alpha 2B [*AMY2B*], glycogen phosphorylase B [*PYGB*], glycogen phosphorylase, muscle associated [*PYGM*], glycogenin 1 [*GYG1*], and alpha‐1,3‐mannosyl‐glycoprotein 4‐beta‐N‐acetylglucosaminyltransferase A [*MGAT4A*]) were positively related to acetic acid‐producing bacteria and four methanogens (Figures 5C and 6). In addition, we found multiple protease genes related to mitochondrial electron respiratory chain, including peroxidase (e.g., glutathione peroxidase 3 [*GPX3*] and peroxiredoxin 6 [*PRDX6*]), coenzyme (e.g., coenzyme Q2 [*COQ2*]), cytochrome c oxidase (COX) (e.g., COX subunit 5A [*COX5A*] and COX subunit 6B1 [*COX6B1*]), and ATPase (e.g., ATP synthase membrane subunit E [*ATP5ME*], ATPase H^+^ transporting V0 subunit a4 [*ATP6V0A4*], ATP synthase F1 subunit epsilon [*ATP5F1E*], and ATPase H^+^ transporting V1 subunit B1 [*ATP6V1B1*]), which were positively correlated with propionic acid‐producing bacteria and negatively correlated with four methanogens (Figures 5C and 6). All these positively and negatively correlated candidate genes were expressed both in rumen tissue and epithelial cells (Figure S12). In brief, we illustrated the potential relationship among host rumen genes, rumen microbiota, and VFAs in rumen methanogenesis. These candidate microbes and genes will provide crucial references for mitigating methane emissions in future studies.

## DISCUSSION

Methane emissions from ruminant livestock contribute a large amount of agricultural greenhouse gas, so a great deal of research has been devoted to finding mitigation strategies. In the past few years, although an increasing number of studies have been conducted to assess the associations among host genetics, rumen microbiota, and methane emission, whether methane emissions could be modulated through the genetic effects of cattle on the ruminal microbiota is still disputed [24, 29, 31]. Moreover, previous studies have not elucidated the relationship between microbes and gene expression in rumen effector organs. Therefore, the host‐microbiome interactions, especially in methanogenesis, remain largely unknown. To better understand this issue, for the first time, we integrated GWAS and TWAS analysis of rumen microbiota in large‐scale population to examine the regulation relationship between host and methanogens. We identified several candidate rumen genes and microbes involved in methanogenesis by combining multiple correlation analyses among host rumen genes, rumen microbes, and VFAs with methanogenic archaea. These preliminary results will provide crucial guidelines for regulating methane emissions through genetic and microbial management strategies.

Heritability estimation helps to understand the extent to which host genetics contributes to phenotypic variation. In this study, we found that the heritability of the rumen core microbiota may be nearly universal, with approximately 70% of rumen taxa exhibiting significant heritability, which is consistent with a recent study in sheep [23]. Previous studies confirmed that CH_4_ production is influenced by host genetics and indicated that methane emissions are moderately heritable, with host genetics able to explain 19%–33% of the phenotypic variation [24, 29, 31]. Our results of heritability estimation shown that the rumen archaea also have significant heritability and the average heritability was 0.41, such as *M. gottschalkii* (*h* ^2^ = 0.47), *M. boviskoreani* (*h* ^2^ = 0.33), and *M. millerae* (*h* ^2^ = 0.27) (Figure 2E,H), which provides important clues for us to search for host genetic markers regulating methanogenic archaeal abundance without directly measuring methane. However, no SNPs passed the significance threshold in the GWAS analysis for methanogens belonging to *Methanobrevibacter* genus. This conclusion is consistent with the findings of several large‐scale cohorts about host genetic influences on the rumen microbiota in cattle [22, 29, 43]. Given that in multiple studies all over the world, no major signal sites associated with methane emissions and methanogenic archaea abundance have been identified. Hence, we speculate that methane production may be a complex phenotype controlled by host microeffect polygenes, only the GWAS method may not be able to find effective genetic markers.

Due to the collected matched transcriptome and microbiome data, this limitation could be addressed by using TWAS, which directly identifies the genes significantly associated with complex traits [32]. In our study, we are the first to construct TWAS on rumen microbiota, and our TWAS detected 28,260 significant gene‐microbe associations (*p* < 3 × 10^−6^), which involved 4652 unique genes associated with 210 microbes. These host rumen epithelial genes provide us with an unprecedented opportunity to study host–microbe interactions in methanogenesis. We further integrated a triple relationship network among host rumen genes, rumen microbiota, and VFAs with methanogens to explore the valuable genes and microbes participating in the methanogenesis pathway. We found that the four archaea species belong to *Methanobrevibacter* genus (e.g., *M. gottschalkii*, *M. boviskoreani*, *M. millerae*, and *M. thaueri*) were positively correlated with acetic acid and A/P ratio but negatively correlated with propionic acid, which are mainly hydrogenotrophic methanogens [10]. During the hydrogenotrophic methanogenesis pathway, methanogens generally convert the fermentation products H_2_ and CO_2_ as substrates to methane [14]. Therefore, to some extent, a promising strategy is to modulate the supply of substrate H_2_ to reduce methane production. In our study, we discovered that some taxa (e.g., *Oscillospirales_UCG‐011*, *Christensenellaceae_R‐7_group*, and *Oscillospiraceae_NK4A214_group*) were positively correlated with four methanogens, acetic acid, and acetic to propionic ratio (A/P), which may be potential acetic acid‐producing bacteria. These acetic acid fermentation microbes produced a large amount of hydrogen (H_2_), which could be used by methanogenic archaea to produce methane. On the contrary, other taxa (e.g., *Prevotella*, *Prevotellaceae_UCG‐003*, and *Anaeroplasma*) were negatively correlated with four methanogens but positively correlated with propionic acid (Figures 5A and 6). Numerous studies have manifested a strongly negative correlation between the abundance of *Prevotella* and methane emissions and suggest that members of the *Prevotella* genus have the ability to utilize hydrogen toward propionic acid production and away from methanogenesis [42, 44, 45].

Our TWAS and correlation analysis results showed that there were significantly related relationships between host rumen epithelial genes and rumen microbiota. The rumen epithelium plays an important role in VFA absorption, metabolism, and H^+^ transport [46, 47]. Generally, the metabolic hydrogen ([H]) in the rumen undergoes reoxidation of reduced cofactors by hydrogenases and also transfers electrons to H^+^ to form H_2_ (molecular hydrogen), which is intercepted by methanogens for the production of methane [40, 48, 49]. Therefore, we speculated that host–microbe interactions in energy metabolism and methane production primarily occur through H^+^ exchange and transport. In our current study, we identified 252 genes associated with rumen fermentation bacteria and methanogenic archaea that were significantly enriched in metabolic pathways. Among these, we observed several positively related genes with acetic acid‐producing bacteria and four methanogens, which enriched in starch and sucrose metabolism pathways (e.g., *AMY2B*, *PYGB*, *PYGM*, *GYG1*, and *MGAT4A*). A previous study found that the starch‐rich diet enriched for amylolytic bacteria, enhanced propionate production through the acrylate pathway with lactate as an intermediate, helping to maintain a healthy rumen and decrease the production of H_2_ available for methanogenesis [50]. We thereby speculate host amylase and glycogen phosphorylase‐related genes could competitively degrade starch and glycogen, which reduces the niche advantage of starch‐utilizing bacteria and increases opportunities for acetate‐producing microorganisms to acquire nutrients. This promotes a shift in rumen fermentation toward acetate‐type fermentation, producing more available hydrogen being used by methanogens to produce methane. In addition, rumen carbohydrates fermentation is accompanied by the metabolism and transport of hydrogen, these H_2_ occurred reversible oxidation under the action of microbial hydrogenases via the reaction H_2_ → 2H^+^ + 2e^−^ [50, 51]. Subsequently, these H^+^ enter the mitochondrial electron respiratory chain of host rumen epithelial cells through the proton gradient and participate in energy metabolism. Furthermore, we discovered a large number of protease genes, such as peroxidase (e.g., *GPX3* and *PRDX6*), coenzyme (e.g., *COQ2*), cytochrome c oxidase (e.g., *COX5A* and *COX6B1*), and ATPase (e.g., *ATP5ME*, *ATP6V0A4*, *ATP5F1E*, and *ATP6V1B1*), which were positively correlated propionic acid‐producing bacteria and negatively correlated with four methanogens (Figure 6). These genes are involved in the mitochondrial respiratory chain for ATP synthesis by mediating electron transfer and proton transport across respiratory chain complexes. The respiratory chain complexes I and III generate superoxide (O_2_^−^) and hydrogen peroxide (H_2_O_2_) from molecular oxygen (O_2_), which is the chief reactive oxygen species (ROS) in mitochondria [52]. The *GPX3* gene and *PRDX6* gene are important peroxisomal enzymes that catalyze the degradation of hydrogen peroxide into water, thereby controlling mitochondrial ROS levels. This process is accompanied by redox reactions and electron transport of NADH/NAD^+^ in complex I [53]. COX is the terminal enzyme of the mitochondrial respiratory chain, reduces oxygen (O_2_) to water, thus contributing to the generation of the electrochemical proton gradient to drive ATP synthesis [54]. *COX5A* and *COX6B1* are the subunits of cytochrome oxidase involved in mitochondrial electron transport and plays a vital protective role in mitochondrial dysfunction, oxidative stress, and cell apoptosis [55, 56]. *ATP5ME*, *ATP5F1E*, *ATP6V0A4*, and *ATP6V1B1* genes are mitochondrial ATP synthase that predominantly utilizes the H^+^ proton gradient for ATP synthesis from ADP and phosphate ions [57]. These above results indicated that host epithelial cells will competitively consume H^+^ to participate in the mitochondrial respiratory chain for energy metabolism, resulting in decreased the availability of substrate hydrogen for methane production (Figure 6). This possible mechanism consistent with the conclusion previously reported that cows with high feed efficiency have a lower abundance of the *Methanobrevibacter* genus [58]. In summary, these findings suggest that host–microbiome interactions in methanogenesis are influenced mainly by hydrogen metabolism. More research will be needed to elucidate these complex regulatory mechanisms in future.

However, there were some limitations of our study. First, the microbiome data were obtained from 16S rRNA gene amplicon sequencing, and microbial genes and functions were not elucidated. Second, the relationship between methanogens and methane emissions is not clear, so the real methane emission data of some individuals should be determined as a prior in future studies. Nevertheless, as the most abundant methanogens, the *Methanobrevibacter* genus was widely suggested to contribute to methane emissions [10]. Notably, previous studies have suggested that propionate‐type fermentation was correlated with low methane emissions, while the acetate‐type fermentation was correlated with high methane emissions [59]. We found that the four methanogens (members of the *Methanobrevibacter* genus) were positively correlated with acetate and negatively correlated with propionate, which suggested a potential link between methane emission and methanogenic archaea abundance. Last but not least, our study used a single cohort, and whether these findings are generally applicable to ruminants need to be verified with different cattle populations and more other ruminant species.

## CONCLUSION

We systematically evaluated the effect of host genetic variants and rumen gene expressions on bovine rumen microbial abundance variation. We found that a more direct relationship between gene expression in rumen effector organs and rumen microbiota abundance. Our results highlight that TWAS is a promising method for determining the host and microbiota associations at gene expression level. By combining multiple relationship networks (genes–taxa–VFAs), we observed that host–microbe interactions in the rumen methanogenesis are primarily involved in substrate hydrogen metabolism and transport. Overall, these findings provide novel insights into the host–microbiome interactions in methanogenesis and offer valuable guidelines for genetic regulation and microbial management strategies to mitigate methane emissions in ruminants.

## METHODS

### Animals and sample collection

A total of 574 male Holstein cattle were selected for this study. All cattle were fattening bulls, approximately 2 years of age, with similar weights, and originated from large‐scale ranches in Ningxia, China. These cattle were fed a similar corn–soybean‐based commercial formula diet and corn silage. All cattle were healthy and fasted for 1 day in the isolation house before slaughtering. The rumen tissues and rumen contents at the ventral sac were collected within 1 h after slaughter. All samples were immediately dipped in liquid nitrogen and then transferred into −80°C freezer until use.

### Genome characterization

The host genome DNA was extracted from rumen tissue samples using the standard phenol–chloroform protocol. The quality of isolated genomic DNA was verified by using 1% agarose gels and DNA concentration was measured by a Qubit DNA Assay Kit in Qubit 3.0 Flurometer (Invitrogen). A total amount of 0.2 μg DNA per sample was used as input material for the DNA library preparations. Briefly, the genomic DNA sample was fragmented by sonication to a size of 350 bp. Then DNA fragments were endpolished, A‐tailed, and ligated with the full‐length adapter for sequencing, followed by further polymerase chain reaction amplification. Subsequently, the DNA libraries were sequenced on the DNBSEQ‐T7 platform (BGI‐Shenzhen) and 150 bp paired‐end reads were generated. Raw FASTQ reads were filtered using fastp (v.0.23.4) [60] software to filter adaptor sequences and low‐quality reads. The clean reads were mapped on the cattle reference genome ARS‐UCD1.2 (GCF_002263795.1) using BWA (v.0.7.17) [61]. We further removed the duplicates using Picard v2.1 (https://broadinstitute.github.io/picard/). The “HaplotypeCaller,” “GenotypeGVCFs,” and “SelectVariants” programs in the Genome Analysis Toolkit (GATK, v.4.1.8.1) [62] were used for SNP calling and genotyping. All raw SNPs were filtered using the “Variant Filtration” module of GATK with the standard parameters “QD < 2.0, MQ < 40.0, FS > 60.0, SOR > 3.0, MQRankSum < −12.5, ReadPosRankSum < −8.0.” Subsequently, only biallelic variants were used to achieve more strict quality control using PLINK (v.1.90b4.6) [63] with the following parameters: SNP call rate >95%, sample call rate > 90%, and minor allele frequency >5%. The remaining SNPs and individuals were used for imputation in Beagle (v.5.4) [64], and the imputed SNPs were refiltered using PLINK with the same above‐described criteria. After these steps, a total of 8,760,052 SNPs distributed across 29 autosomes and 574 individuals were obtained for subsequent analyses.

### Microbiome characterization

Microbial DNA from rumen content samples was extracted using the QIAamp Fast DNA Stool Mini Kit (Qiagen) following the manufacturer's instructions. The V3–V4 region of the 16S rRNA gene for bacteria was amplified using specific primers 341F (CCTAYGGGRBGCASCAG) and 806 R (GGACTACNNGGGTATCTAAT). The V6–V8 region of the 16S rRNA gene for archaea was amplified using specific primers 915 F (AGGAATTGGCGGGGGAGCAC) and 1386 R (GCGGTGTGTGCAAGGAGC). All PCR reactions were carried out with 15 μL of Phusion High‐Fidelity PCR Master Mix (New England Biolabs), 0.2 μM of forward and reverse primers, and about 10 ng template DNA. Thermal cycling consisted of initial denaturation at 98°C for 1 min, followed by 30 cycles of denaturation at 98°C for 10 s, annealing at 50°C for 30 s, and elongation at 72°C for 30 s. Finally, 72°C for 5 min. Sequencing libraries were generated using TruSeq DNA PCR‐Free Sample Preparation Kit (Illumina) following manufacturer's recommendations and index codes were added. The library quality was assessed on the Qubit 2.0 Fluorometer (Thermo Fisher Scientific) and Agilent Bioanalyzer 2100 system. At last, the library was sequenced on NovaSeq 6000 platform (Illumina), and 250 bp paired‐end reads for bacteria and 300 bp paired‐end reads for archaea were generated. The raw paired‐end sequences were demultiplexed, quality filtered with fastp (v.0.23.4) [60], and merged into tags using FLASH (v.1.2.7) [65]. High‐quality reads were selected after quality control, denoising, and chimera removal using the DADA2 [66] plugin in QIIME2 (v.2022.8) [67] under the recommended parameters to generate ASVs. ASVs identified as chloroplasts and mitochondria were excluded. A total of 5974 and 3588 ASVs for bacteria and archaea remained after quality control. The taxonomic classification of the ASVs was performed using the SILVA (v.138) database [68]. Only taxa with average relative abundance > 0.001% and with a prevalence > 20% were included in the downstream analysis. Before diversity analysis, the number of bacteria and archaea sequences per sample was rarefied to a sampling depth of 10,000 and 5000, respectively. ⍺ Diversity metrics including Chao1, ACE, observed_features, Shannon, and Simpson indices were calculated using QIIME2 software. β Diversity (principal coordinates analysis) was calculated to determine differences in the microbial community structure based on the Bray–Curtis distances matrices. The relative abundance of each microbial taxon was log_10_‐transformed in subsequent analysis.

### Transcriptome characterization

Total RNA was extracted from rumen tissues using TRIzol reagent (Invitrogen) according to the manufacturer's instructions. Due to the degradation of RNA in some samples, the 460 high‐quality RNA samples were used to construct sequencing libraries. After library quality control, the libraries were sequenced using DNBSEQ‐T7 platform (BGI), and 150 bp paired‐end reads were generated. Raw FASTQ data were first checked for quality using fastp (v.0.23.4) [60] software with default parameters. Then the clean reads were mapped to the cattle reference genome ARS‐UCD1.2 using HISAT2 (v.2.2.1) [69] software. Transcript assembly and standardized transcripts per kilobase million (TPM) values were obtained using StringTie (v.2.1.2) [70] to construct the gene expression matrix. To reduce the amount of computation and increase the statistical power, we kept genes with TPM > 0.1 in ≥20% of samples and any outlier individuals (out of mean ± 3 SD) were removed. Finally, a total of 454 rumen samples and 16,674 genes were used for subsequent analysis.

### Co‐occurrence network of rumen microbiota

To understand microbial interactions in the rumen, we constructed co‐occurrence networks based on the log_10_‐transformed relative abundance of each microbial taxon. The taxon correlation network was calculated by Spearman's correlation coefficient in the R package Hmisc (v.4.6.0). Only the genus‐level taxa with prevalence >50% were used, and only those with a correlation coefficient >0.5 or <−0.5 and Benjamini–Hochberg (BH) adjusted *p* value < 0.05 were displayed. Interaction networks were then visualized using Cytoscape (v.3.8.2) [71] and Gephi (v.0.10) [72] software.

### 16S rRNA gene function prediction using PICRUSt2

Functional pathway analysis of the 16S rRNA gene sequencing data obtained from all ASVs was performed using PICRUSt2 [73] to infer the metagenomes. PICRUSt2 predictions based on several gene family databases are supported by default, including Kyoto Encyclopedia of Genes and Genomes (KEGG) orthologs and Enzyme Commission numbers. In this study, the abundance of pathways was normalized into CPM, and only the pathways with CPM > 5 in at least 20% of samples remained.

### Investigation of the association between host genetics and the rumen microbiota

To clarify the association between host genetic kinship and microbial composition similarity based on pairs of individuals, we first calculated the genetic relationship matrix based on all SNP genotypes using GCTA (v.1.94.1) [74], and then transformed it to the kinship matrix using R script. The microbial composition similarity (1 − dissimilarity) was measured by the Bray–Curtis distance of all ASV counts using the vegan package in R. We evaluated the correlation between host genetic kinship and microbiome similarity through Spearman's rank correlation *ρ* using Mantel tests with 999 permutations. However, considered that most of the host genetic relatedness between individuals was weak, which may affect the estimated reliability. Therefore, in the current study, pairs of individuals with an estimated genetic kinship ≤0.05 and ≥0.10 were considered genetically more distant and close relatives, respectively [75, 76]. In total, 164,451 pairs of individuals ($C_{574}^{2}$) were included in our study, and we randomly selected 500 pairs of more distant relatives and 500 pairs of closer relatives from this data set using the dplyr package in R. Subsequently, the correlations between the host genetic kinship and microbiome similarity of these pairs of individuals were recalculated.

### Heritability and expressability estimation

The microbiome heritability of single‐taxon at different levels was estimated by the genome‐based restricted maximum likelihood method implemented in GCTA [74] to estimate the microbial variance explained by all SNPs. Narrow‐sense heritability was estimated as *h* ^2^ = $\sigma_{\text{g}}^{2}/\sigma_{\text{p}}^{2}$, where $\sigma_{\text{g}}^{2}$ and $\sigma_{\text{p}}^{2}$ are the genetic variance and phenotypic variance, respectively. Similarly, the “expressability” (*e* ^2^) was used to estimate the microbial variance explained by all genes. First, all gene TPM values were normalized and used to construct the gene ERM using OSCA (v.0.46.1) [77] software, following the command *osca ‐‐befile myprofile ‐‐make‐orm ‐‐out myorm*. Next, we use an OREML module in OSCA to estimate the variance explained by the genes (*e* ^2^), following the command *osca ‐‐reml ‐‐reml‐alg 0 ‐‐orm myorm ‐‐pheno my.phen ‐‐qcovar my.qcovar ‐‐out myreml*. The microbial variance explained by the gene expression variance was estimated as *e* ^2^ = $\sigma_{\text{e}}^{2}/\sigma_{\text{p}}^{2}$, where $\sigma_{\text{e}}^{2}$ and $\sigma_{\text{p}}^{2}$ are the gene expression variance and phenotypic variance, respectively. *p* Values for the heritability and expressability estimates were computed using the likelihood ratio test, and *p* < 0.05 was considered to have significant heritability and expressability.

### mbGWAS

Because of the microbiome data that have the characteristic of zero‐inflated, which often affects the accuracy of the analysis model [78]. In our study, we first defined the taxa that presented in >80% of individuals as quantitative traits (relative abundance), and those presented in 20%–80% of individuals as binary traits (presence/absence), while taxa presented in less than 20% of individuals were discarded from further analyses. Then we performed mbGWAS analysis for relative abundance or presence/absence traits of taxa using a linear mixed model in GEMMA (v.0.98.5) [79]: *y* = *Wα* + *Xβ* + *u* + *e* (where *y* is a vector of phenotypes; *W* is a *n *× *p* matrix of covariates (fixed effects), including the top five host genetic principal components; *α* is a *p*‐vector of effects for the covariates including the intercept; *X* is an *n*‐vector of marker genotypes; and *β* is the effect size of the marker and is an estimate of the marker additive effect. *u* is a vector of random effects that follows the normal distribution N (0, *G* $\sigma_{\text{g}}^{2}$), where *G* is the *n *× *n* genetic kinship matrix and $\sigma_{\text{g}}^{2}$ is the polygenic additive variance. *e* is a vector of residual errors). The Bonferroni corrected *p* values of the SNP effects were calculated for significant associations. The genome‐wide significance threshold was set at 5.71 × 10^−9^ (0.05/8,760,052), and the suggestive significance threshold was set at 1.14 × 10^−7^ (1/8,760,052). The Manhattan plots and Q–Q plots were generated using the qqman and CMplot packages in the R. The identified SNPs were annotated using ANNOVAR [80]. To demarcate independent association signals across the putative regions, we performed a linkage disequilibrium (LD) estimation by applying LD pruning on the SNP data using PLINK (command ‐‐*indep‐pairwise* 1000 10 0.2), pairs of SNPs with *r* ^2^ greater than 0.2 were regarded as highly linked.

### Comparison of the association between rumen gene expression and rumen microbiota

To further confirm the association between gene expression relationship and microbial composition similarity based on 454 pairs of individuals. The gene ERM of all genes and TWAS significantly associated genes were constructed, respectively. We then evaluated the correlation between the gene ERM and microbiome similarity matrix through Spearman's rank correlation *ρ* using Mantel tests with 999 permutations.

### mbTWAS

For mbTWAS, a total of 454 paired samples with both transcriptome and microbiome data were selected, which included 16,674 genes and 317 taxa for analysis. The associations between log_10_‐transformed gene expression and microbial relative abundance were calculated by linear regression of the EWAS program in OSCA (v.0.46.1) [77] and PrediXcan (v.0.9.1) [81] software. Briefly, the first step is to read the gene expression data and save it in binary format with the following parameters: *osca ‐‐efile myprofile.txt ‐‐gene‐expression ‐‐make‐bod ‐‐out myprofile*. Next, the probe information is sometimes not available in the original gene expression data. These informations can be updated using the command *osca ‐‐befile myprofile ‐‐update‐opi annotated.opi*. Finally, the linear regression analysis model was used to associate the gene expressions and taxa abundance phenotypes with the parameters: *osca ‐‐befile myprofile ‐‐pheno my.phen ‐‐qcovar my.qcovar ‐‐linear ‐‐out my*. In our test, we found that the results of OSCA software could fully include the results from PrediXcan software, so we selected all the results obtained by OSCA software. The Bonferroni correction was used to claim transcriptome‐wide significance (*p* = 0.05/16,674 = 3 × 10^−6^).

### *Cis*‐eQTL mapping for rumen gene expression

We conducted *cis*‐eQTL mapping for the rumen gene expression phenotype with 454 individuals. We kept genes with TPM > 0.1 in ≥20% of samples and normalized gene expression values by log_10_‐transformed. Then the *cis*‐eQTL mapping was performed using a linear regression model, implemented in FastQTL (v.2.184) [82], while accounting for the top five genotype PCs as estimated covariates. We defined the *cis*‐window of genes as ±1 Mb of transcriptional start site to test associations of genes with genetic variants. We first conducted *cis*‐eQTL mapping in a permutation mode with the command *‐‐permute 1000 10000*, to calculate empirical *p* values and used the FDR method to correct the β‐approximated and empirical *p* values for multiple testing. We identified genes with at least one significant *cis*‐eQTL (FDR < 0.05) as eGene. We then applied the nominal mode to identify a list of significant eGene‐eSNP pairs. We defined the empirical *p* value of the eGene that was closest to an FDR of 0.05 as genome‐wide min *p* value threshold (*pt*). The gene‐level threshold for each gene was obtained by *qbeta* (*pt*, *beta_shape1*, *beta_shape2*) in R software, where *beta_shape1* and *beta_shape2* resulted in fastQTL with permutation mode. We then considered SNPs with a nominal *p* value below the min gene‐level threshold as significant *cis*‐eQTL.

### Colocalization of *cis*‐eQTL and GWAS loci

To detect the shared variants of rumen gene expression and rumen microbial phenotype, we first simply overlapped 15,301 significant GWAS loci (*p* < 1 × 10^−5^) with 416,109 eSNPs tested in the eQTL mapping analysis, resulting in 770 common loci. We further conducted colocalization analysis using Coloc (v.5.2.2) [83] with the *coloc.abf ()* function. We obtained posterior probability values for the H4 case (PP.H4); that is, both traits are associated and share a single causal variant. We considered the SNP.PP.H4 with the 95% credible set were considered as more likely causal variants for downstream analysis.

### Determination of VFAs in rumen contents

For the VFA measurements, the rumen fluid was centrifuged at 13,000*g* for 10 min at 4°C. Next, the 1 mL supernatant was mixed with 0.2 mL of 25% metaphosphoric acid and then centrifuged for 15 min at 10,000*g* at 4°C. Following this, 2 mL of the supernatant was mixed with 200 μL of crotonic acid and then the supernatant was filtered into a sample bottle through a 0.45 μm filter. The VFAs were separated and quantified using gas chromatography (Agilent 7890B system) with a polar capillary column (DB‐FFAP, 30 m × 0.32 mm × 0.25 μm) and a flame ionization detector. The injector and detector temperatures were set at 200°C and 250°C, respectively. The column temperature was increased from 45°C to 150°C at a 20°C/min ramp and then held for 5 min [84].

### Functional pathway enrichment analysis of gene sets

The online tools KOBAS‐i (http://kobas.cbi.pku.edu.cn/) [85] and the OmicShare tools (https://www.omicshare.com/tools) [86] were used to identify the significantly enriched KEGG pathways for all gene sets in this study. Significance was calculated using the hypergeometric test/Fisher's exact test, and a *p* < 0.05 was considered.

### Statistical analysis

Statistical analysis was performed using the R packages (v.4.3.1) and GraphPad Prism 9 (GraphPad Software). The difference analysis between the two groups was compared using the student's *t* test, and the results are expressed as mean ± SEM. The correlations between the rumen genes, microbial taxa, and VFAs were detected by the Spearman correlation test using the “corr.test” function in the R package “psych” and *p* values were adjusted using the BH method. A *p* value of less than 0.05 was considered significant for all tests.

## AUTHOR CONTRIBUTIONS

Yu Wang and Shengru Wu conceived the project and designed the research. Wei Wang, Zhuohui Li, and Zhenyu Wei performed the majority of the data analysis. Wei Wang, Zhenyu Wei, Zhuohui Li, Yanliang Song, Anguo Liu, Xinmei Li, Manman Li, and Huimei Fan participated in the sample collection. Jianrong Ren measured the volatile fatty acids in the rumen fluid. Wei Wang, Zhuohui Li, and Jingyi Xu generated the figures. Liangliang Jin provided the cattle rumen single‐cell gene expression data. Wei Wang wrote the first draft of the manuscript. Zhannur Niyazbekova, Wen Wang, Yuanpeng Gao, Yu Jiang, Junhu Yao, Fuyong Li, Shengru Wu, and Yu Wang revised the manuscript. All authors have read the final manuscript and approved it for publication.

## CONFLICT OF INTEREST STATEMENT

The authors declare no conflict of interest.

## ETHICS STATEMENT

The ethics application (DK2022020) was approved by the Animal Ethical and Welfare Committee of Northwest A&F University.

## Supporting information


**Figure S1.** Data characteristics of 16S rRNA gene sequencing of rumen microbiota.
**Figure S2**. Spearman correlations between the rumen microbiota and the predicted KEGG pathways of the 16S rRNA gene.
**Figure S3**. Individual genetic information of 574 Holstein cattle.
**Figure S4**. The genomic inflation factor (*λ*_GC_) for mbGWAS analysis of 317 phenotypes.
**Figure S5**. Microbial genome‐wide association studies.
**Figure S6.** Colocalization of GWAS loci and *cis*‐eQTLs.
**Figure S7**. The TWAS correlation network of rumen microbiota and rumen genes.
**Figure S8.** The overlapping genes of cis‐eQTL gene, GWAS nearest gene, and TWAS gene.
**Figure S9.** Characteristics of TWAS analysis using different sample sizes.
**Figure S10.** Correlation analysis between 4 rumen archaea (*Methanobrevibacter* genus) and 36 rumen bacteria.
**Figure S11.** The KEGG pathway enrichment analysis of 40 taxa TWAS genes.
**Figure S12.** The expression of candidate genes in bovine rumen snRNA‐seq and bulk RNA‐seq data.

**Table S1**. The results of metadata annotation of 5,974 ASVs for bacteria.
**Table S2.** The results of metadata annotation of 3,588 ASVs for archaea.
**Table S3**. The average relative abundance and prevalence of cattle rumen microbiota.
**Table S4**. Functional annotation of genome‐wide genetic variants.
**Table S5**. Heritability estimation of 317 single taxon.
**Table S6**. Results summary of the independent loci of genome‐wide significant SNPs associated with rumen microbial taxa.
**Table S7**.The overlapped SNPs between GWAS loci and *cis*‐eQTLs.
**Table S8.** Expressability estimation of 317 single taxon.
**Table S9.** Rumen fermentation parameters of Holstein cattle.
**Table S10**. Correlation analysis between microbial taxa and volatile fatty acids (VFAs).
**Table S11.** Correlation analysis between rumen genes and microbial taxa.

## Data Availability

The raw sequencing data used and described in this study have been deposited into CNGB Sequence Archive (CNSA) (https://db.cngb.org/cnsa/) of China National GeneBank DataBase (CNGBdb) with accession numbers CNP0003615 and CNP0005690. The data and scripts used are saved in GitHub (https://github.com/WeiWang-NWAFU/Host-microbiome-iMeta2024). More detailed data information can be obtained by contacting the corresponding author. Supporting Information (figures, tables, scripts, graphical abstract, slides, videos, Chinese translated version, and update materials) may be found in the online DOI or iMeta Science https://www.imeta.science/.
