# Supplementary material for: Integrating genome‐ and transcriptome‐wide association studies to uncover the host–microbiome interactions in bovine rumen methanogenesis
Source: Imeta. 2024 Sep 3;3(5):e234. doi: 10.1002/imt2.234 (PMC11487568; doi:10.1002/imt2.234)
Supplement: Supplementary file 1 — Figure S1. Data characteristics of 16S rRNA gene sequencing of rumen microbiota. Figure S2. Spearman correlations between the rumen microbiota and the predicted KEGG pathways of the 16S rRNA gene. Figure S3. Individual genetic information of 574 Holstein cattle. Figure S4. The genomic inflation factor (λ GC) for mbGWAS analysis of 317 phenotypes. Figure S5. Microbial genome‐wide association studies. Figure S6. Colocalization of GWAS loci and cis‐eQTLs. Figure S7. The TWAS correlation network of rumen microbiota and rumen genes. Figure S8. The overlapping genes of cis‐eQTL gene, GWAS nearest gene, and TWAS gene. Figure S9. Characteristics of TWAS analysis using different sample sizes. Figure S10. Correlation analysis between 4 rumen archaea (Methanobrevibacter genus) and 36 rumen bacteria. Figure S11. The KEGG pathway enrichment analysis of 40 taxa TWAS genes. Figure S12. The expression of candidate genes in bovine rumen snRNA‐seq and bulk RNA‐seq data. [file IMT2-3-e234-s001.docx]

**Supporting Information to**

**Integrating genome- and transcriptome-wide association studies to uncover host-microbiome interactions in bovine rumen methanogenesis**

**Running title: Host-microbiome interactions in bovine rumen methanogenesis**

Wei Wang^1#^, Zhenyu Wei^1#^, Zhuohui Li^1#^, Jianrong Ren^2^, Yanliang Song^3^, Jingyi Xu^2^, Anguo Liu^1^, Xinmei Li^1^, Manman Li^1^, Huimei Fan^1^, Liangliang Jin^1^, Zhannur Niyazbekova^1^, Wen Wang^4^, Yuanpeng Gao^3,5^, Yu Jiang^1,5^, Junhu Yao^2,5^, Fuyong Li^6*^, Shengru Wu^2,5*^, Yu Wang^1,5*^

^1^Department of Animal Genetics, Breeding and Reproduction, College of Animal Science and Technology, Northwest A&F University, Yangling, China

^2^Department of Animal Nutrition and Environmental Health, College of Animal Science and Technology, Northwest A&F University, Yangling, China

^3^Department of Clinical Veterinary, College of Veterinary Medicine, Northwest A&F University, Yangling, China

^4^School of Ecology and Environment, Faculty of Life Sciences and Medicine, Northwestern Polytechnical University, Xi’an, China

^5^Key Laboratory of Livestock Biology, Northwest A&F University, Yangling, China

^6^Department of Animal Science and Technology, College of Animal Sciences, Zhejiang University, Hangzhou, China

^#^These authors contributed equally: Wei Wang, Zhenyu Wei, and Zhuohui Li

^*^Correspondence: fuyong@zju.edu.cn (Fuyong Li), wushengru2013@nwafu.edu.cn (Shengru Wu), and wang_yu@nwafu.edu.cn (Yu Wang)

**Supplementary Figures**

**
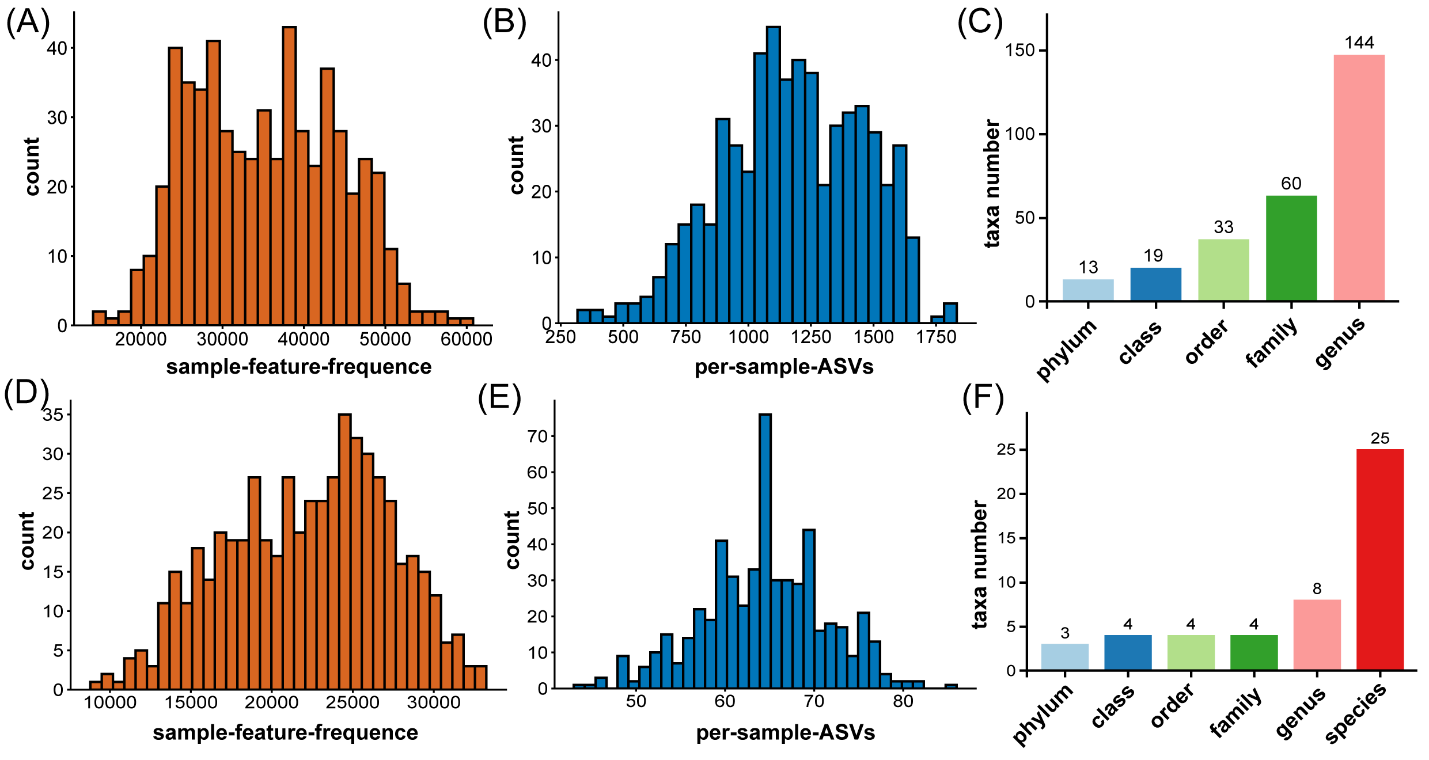
**

**Figure S1.** Data characteristics of 16S rRNA gene sequencing of rumen microbiota. (A) and (D) High quality feature sequence counts of each sample for bacteria and archaea. (B) and (E) The ASV counts of each sample for bacteria and archaea. (C) The results of taxonomic annotation at five levels (from phylum to genus) for bacteria (prevalence > 20%). (F) The results of taxonomic annotation at six levels (from phylum to species) for archaea (prevalence > 20%).

**
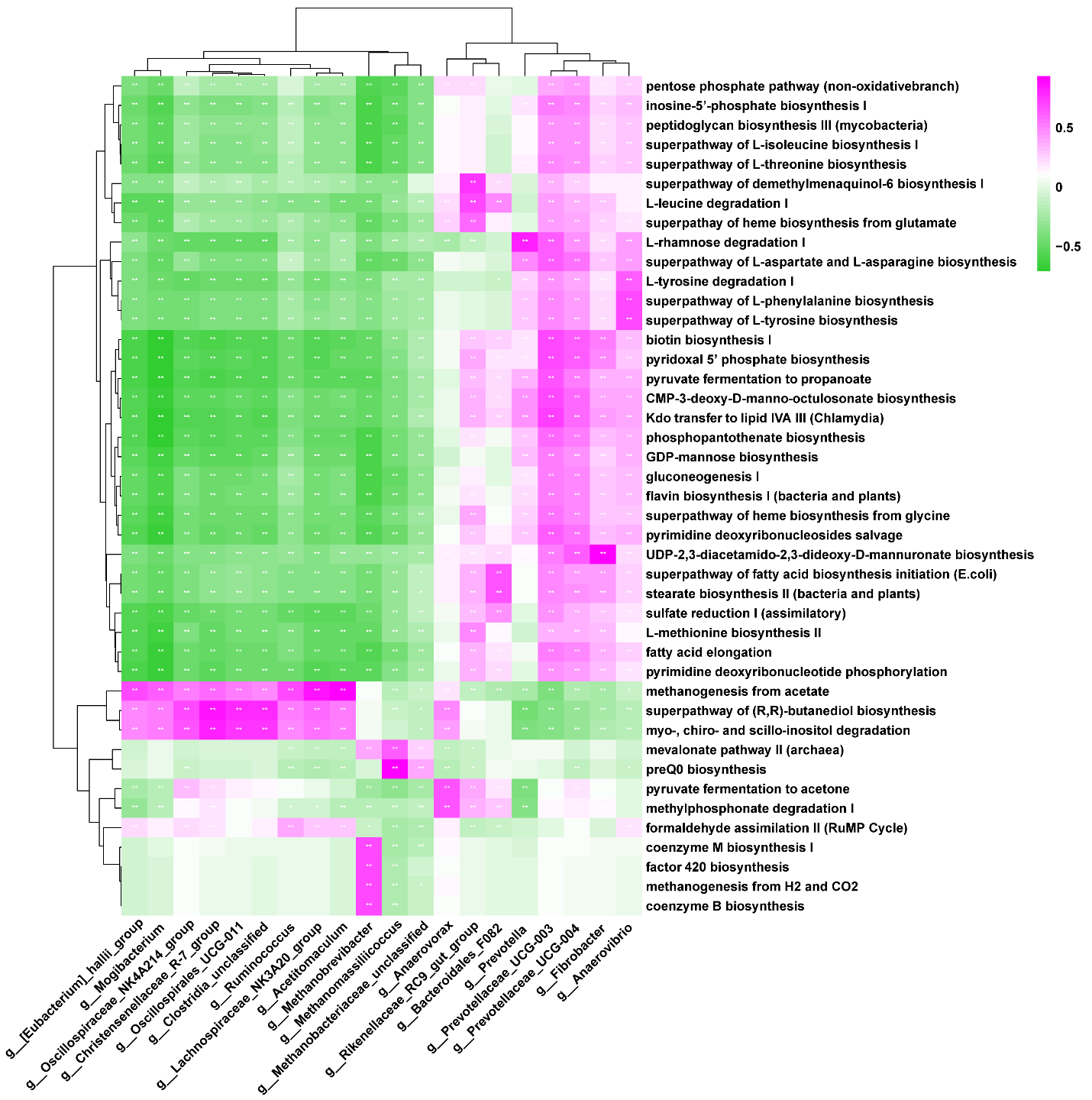
**

**Figure S2.** Spearman correlations between the rumen microbiota and the predicted KEGG pathways of the 16S rRNA gene. Correlation coefficients |r| > 0.5 and adjusted *p* values < 0.05 are represented.


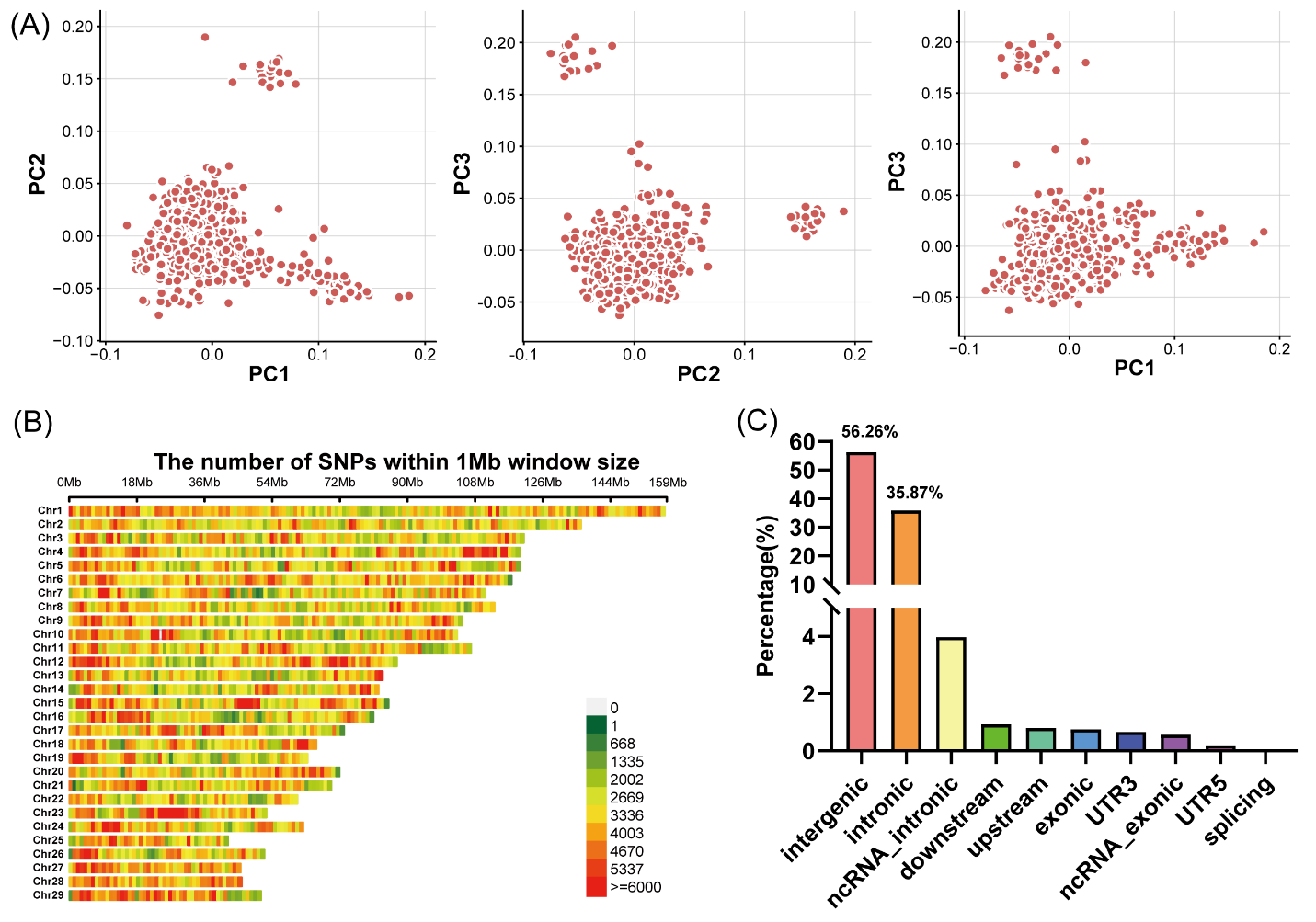


**Figure S3.** Individual genetic information of 574 Holstein cattle. (A) The SNP-based population structure using principal component analysis. Genome-wide genetic variants distribution on 29 autosomes (B) and functional annotation (C) of 574 Holstein cattle.

**
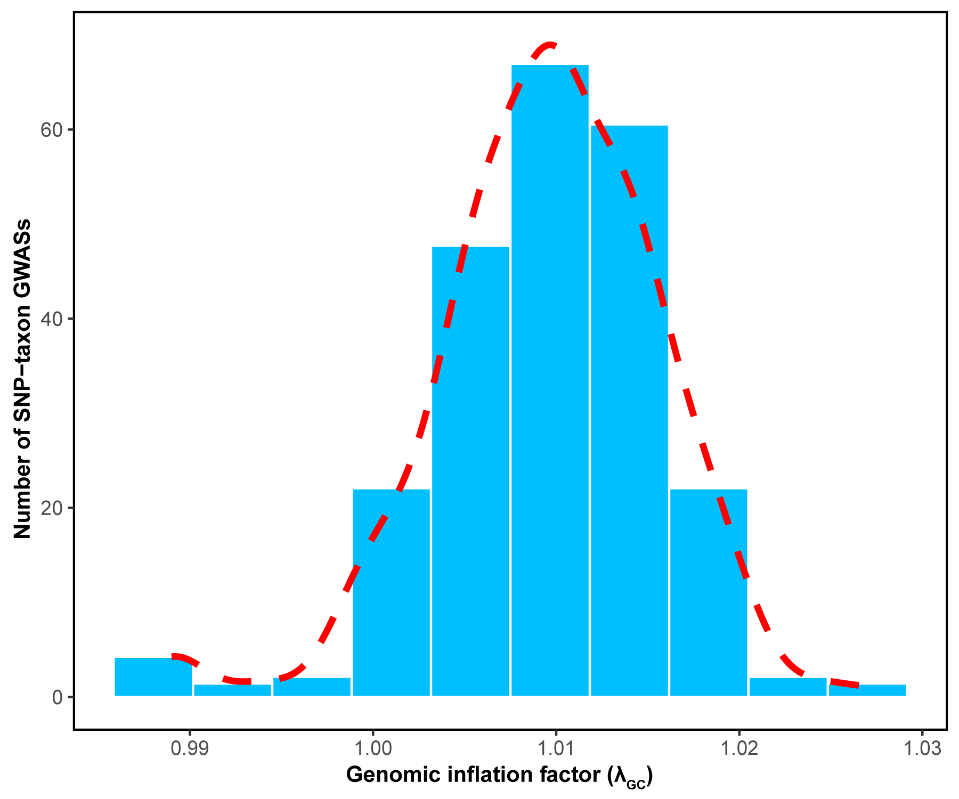
Figure S4.** The genomic inflation factor (λ_GC_) for mbGWAS analysis of 317 phenotypes.


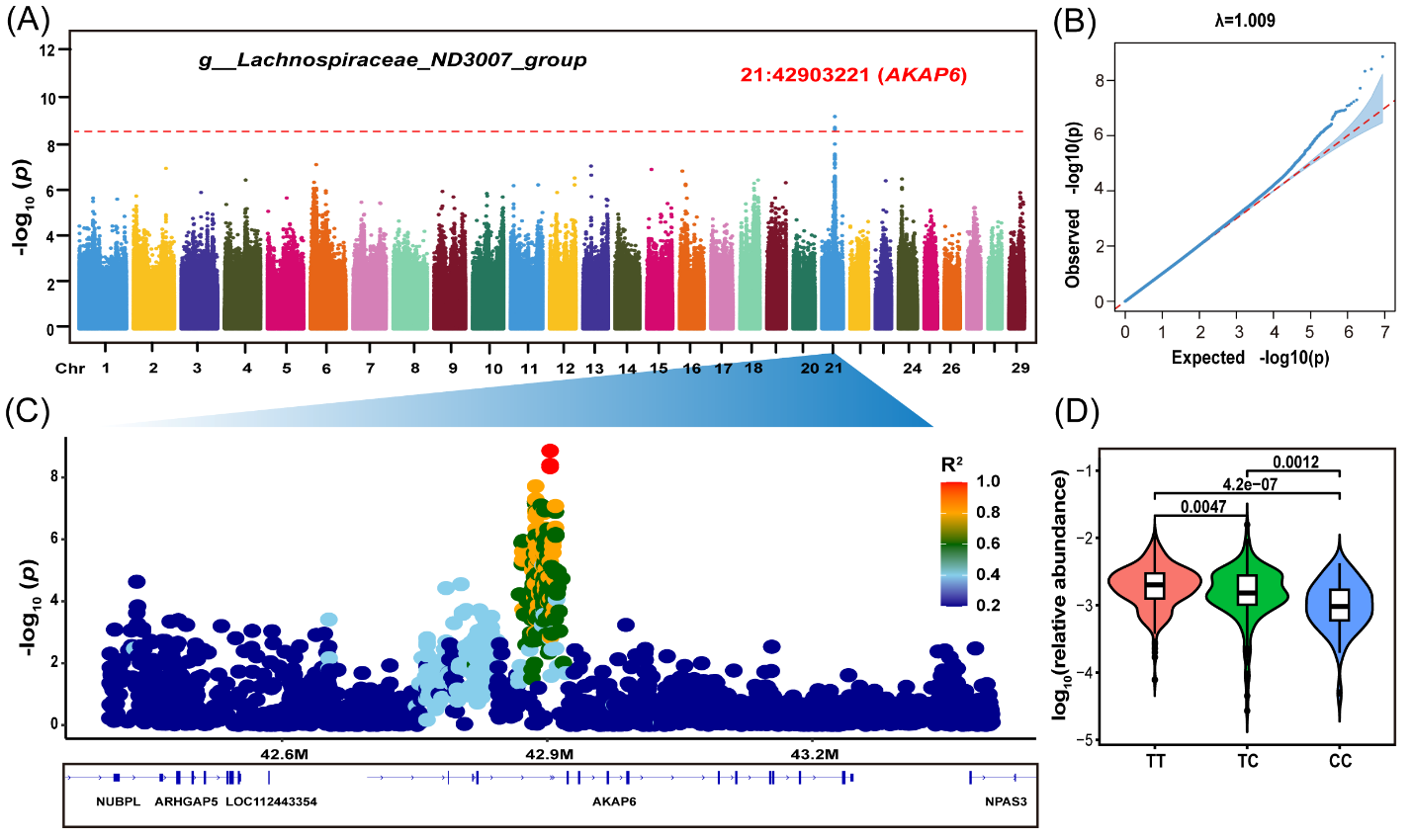


**Figure S5.** Microbial genome-wide association studies. (A) Genome-wide Manhattan plot for *Lachnospiraceae_ND3007_group* abundance trait. (B) Quantile-quantile (Q-Q) plot of the *p* values. (C) Close-up plot of a 1Mb window around the SNP (chr21:42903221, *p* = 1.25×10^-9^) with the highest association. (D) The violin plots of *Lachnospiraceae_ND3007_group* normalized abundance with each genotype at the most strongly associated SNP (chr21:42903221).


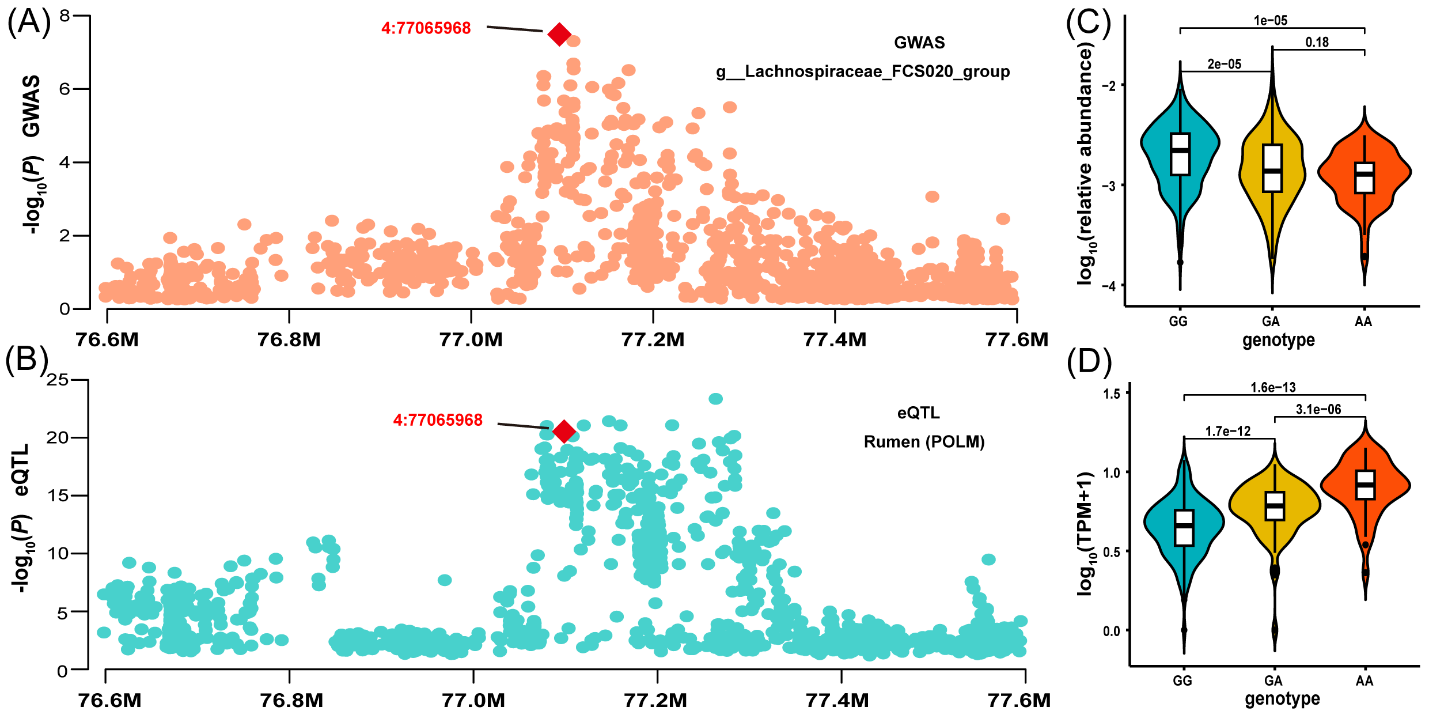


**Figure S6.** Colocalization of GWAS loci and *cis*-eQTLs. (A) and (B) Example of colocalization of a *cis*-eQTL of the *POLM* gene in rumen and the GWAS loci of *Lachnospiraceae_FCS020_group* abundance trait in cattle on chromosome 4. The Manhattan plot shows the variations within the ± 0.5 Mb region of significant site (chr4:77065968). (C) The violin diagram shows the difference of *Lachnospiraceae_FCS020_group* abundance with each genotype at the colocalized SNP (chr4:77065968). (D) The violin diagram shows the difference of *POLM* gene expression with each genotype at the colocalized SNP.


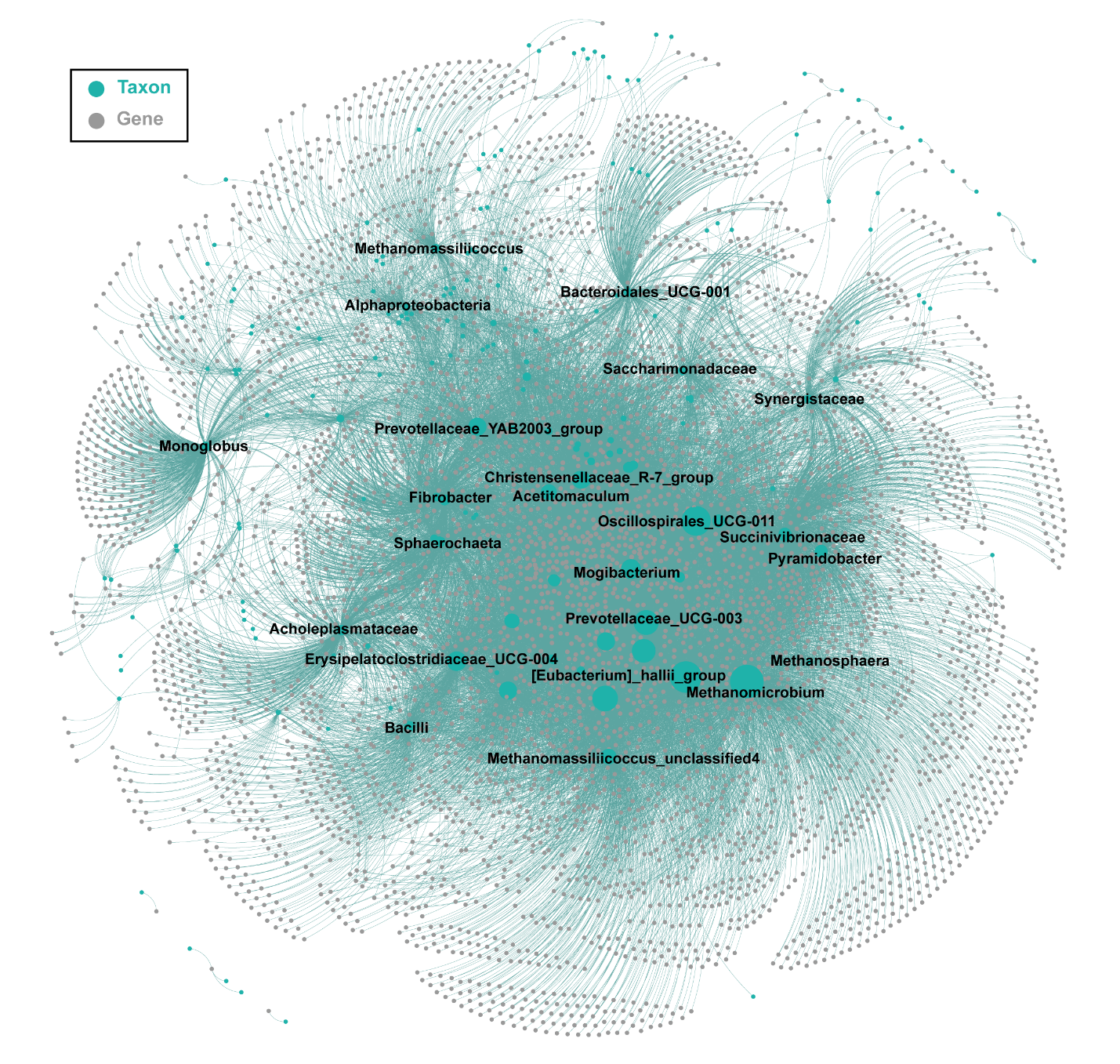


**Figure S7.** The TWAS correlation network of rumen microbiota and rumen genes. This network based on the 28,260 significant gene-taxon pairs of TWAS (*p* < 3×10^-6^), which involved 210 taxa and 4652 unique genes.

**
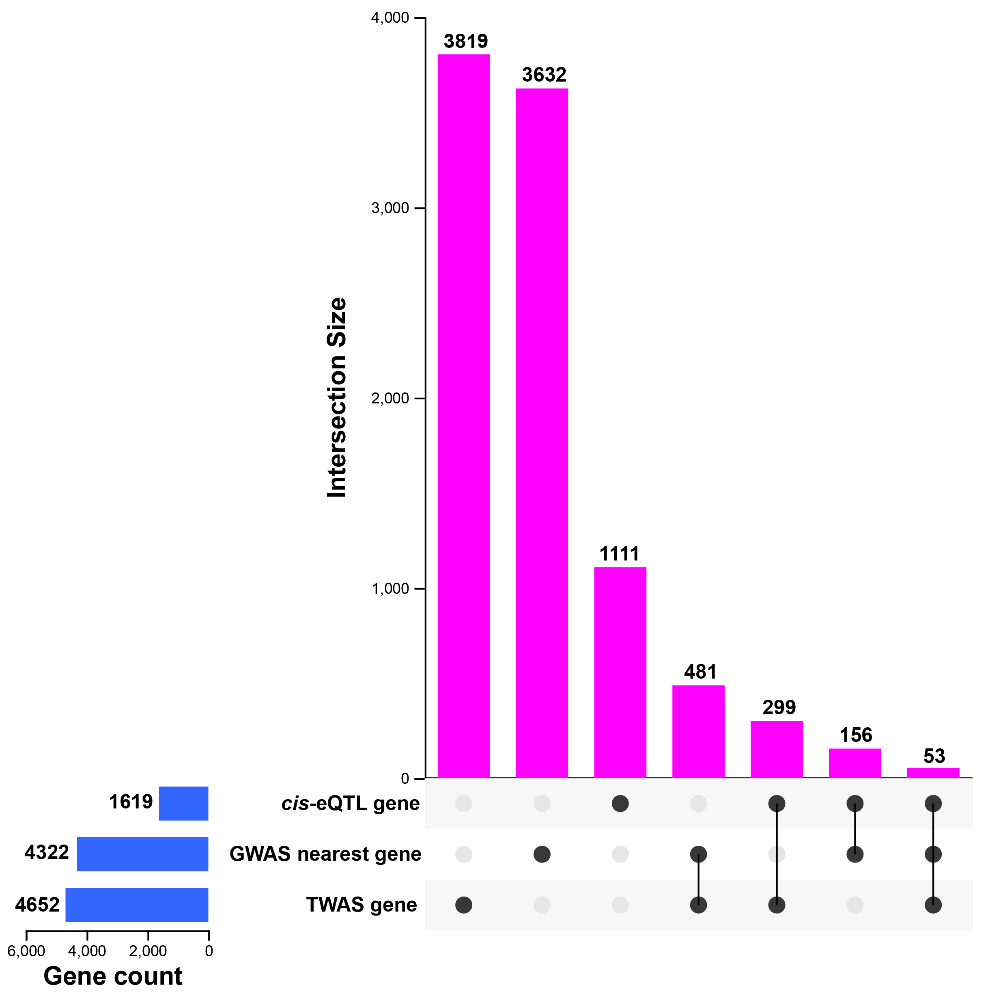
Figure S8.** The overlapping genes of *cis*-eQTL gene, GWAS nearest gene, and TWAS gene.


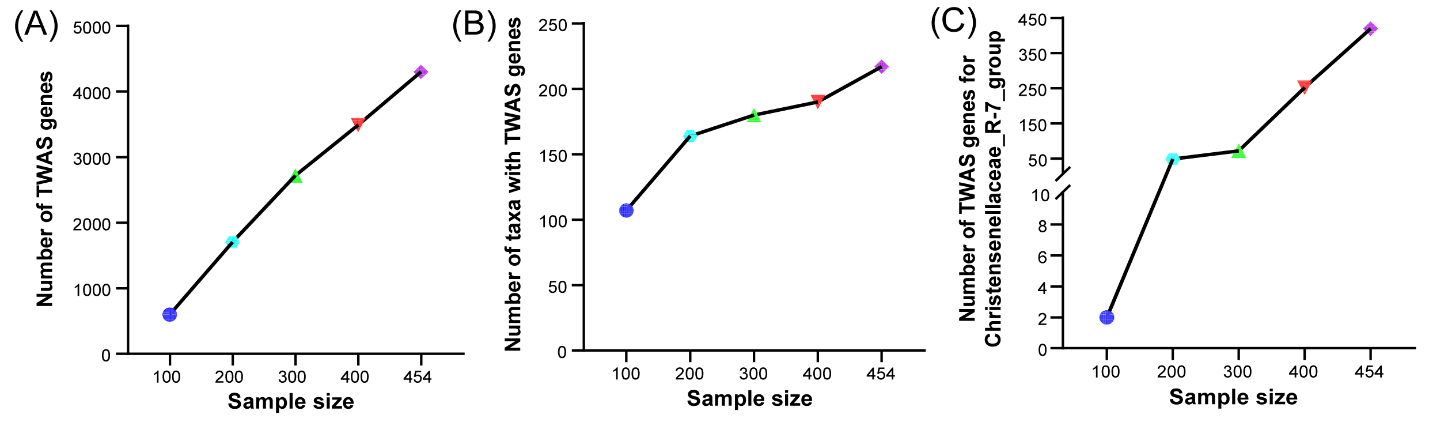


**Figure S9.** Characteristics of TWAS analysis using different sample sizes. (A) The number of significant TWAS genes of all tested taxa under different sample sizes. (B) The number of taxa with significant TWAS genes using different sample sizes. (C) The number of significant TWAS genes of *Christensenellaceae_R-7_group* under different sample sizes.

**
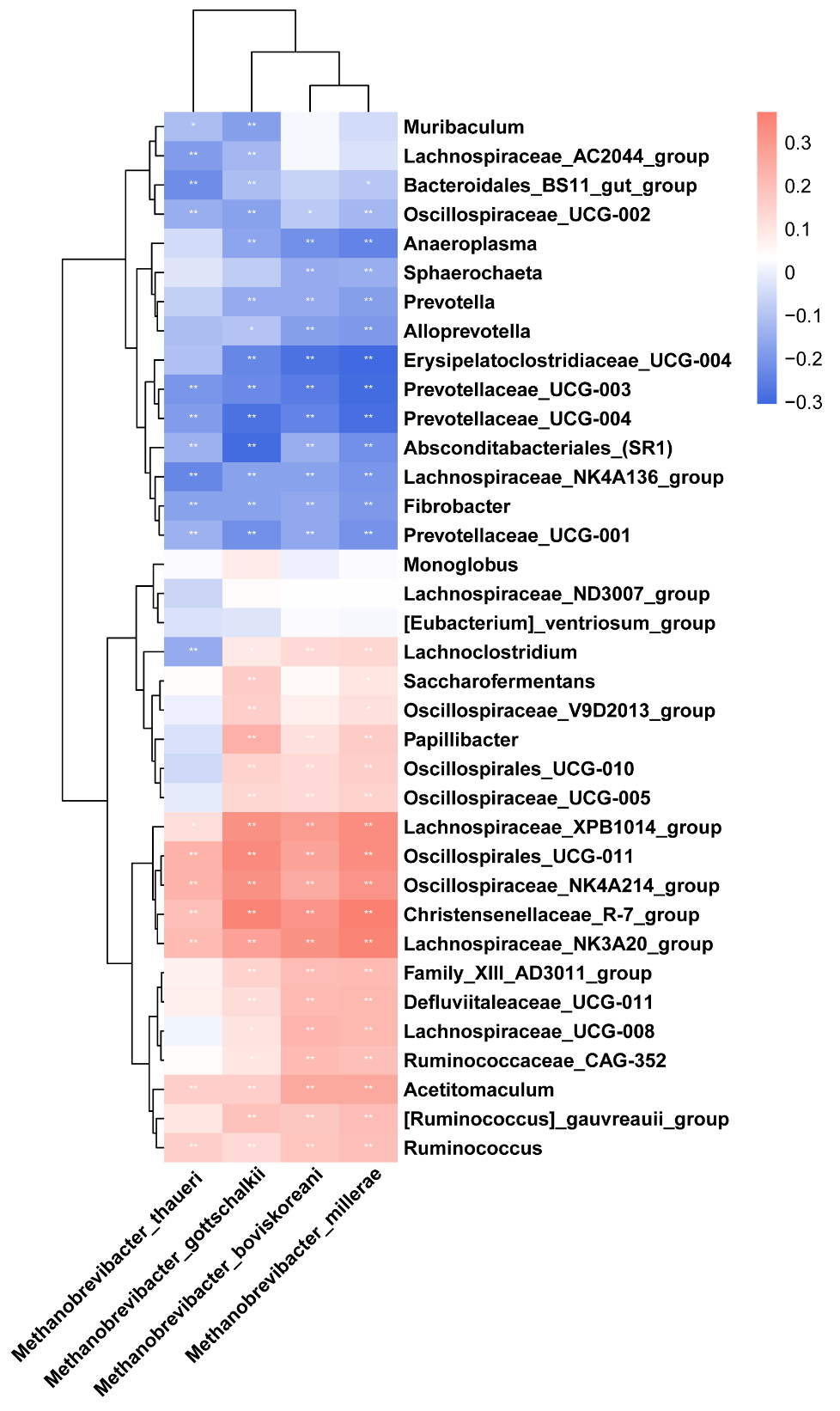
Figure S10.** Correlation analysis between 4 rumen archaea (*Methanobrevibacter* genus) and 36 rumen bacteria with correlation coefficients |r| > 0.2. * represents *p* < 0.05, ** represents *p* < 0.01.


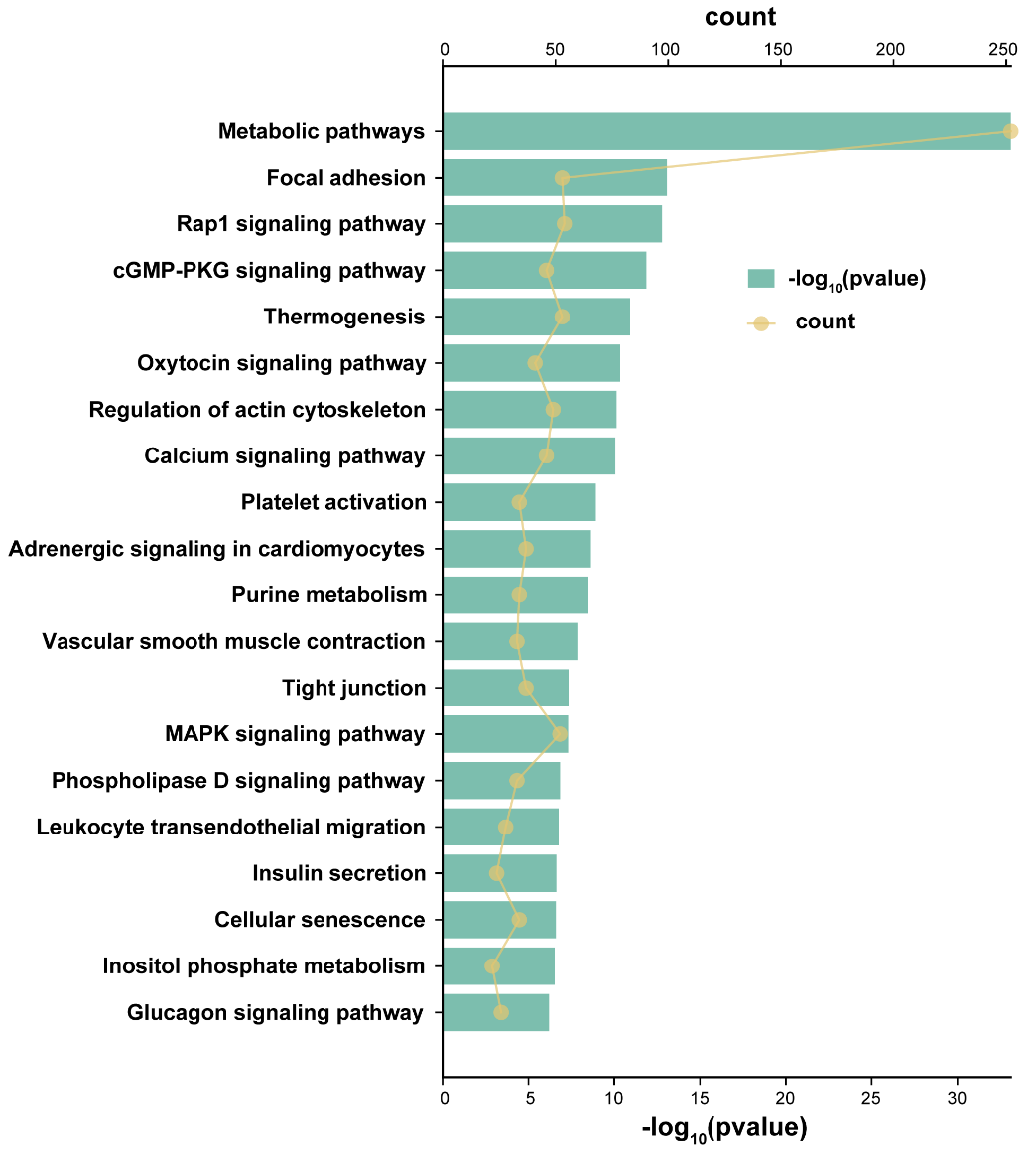
**Figure S11.** The KEGG pathway enrichment analysis of 40 taxa TWAS genes.


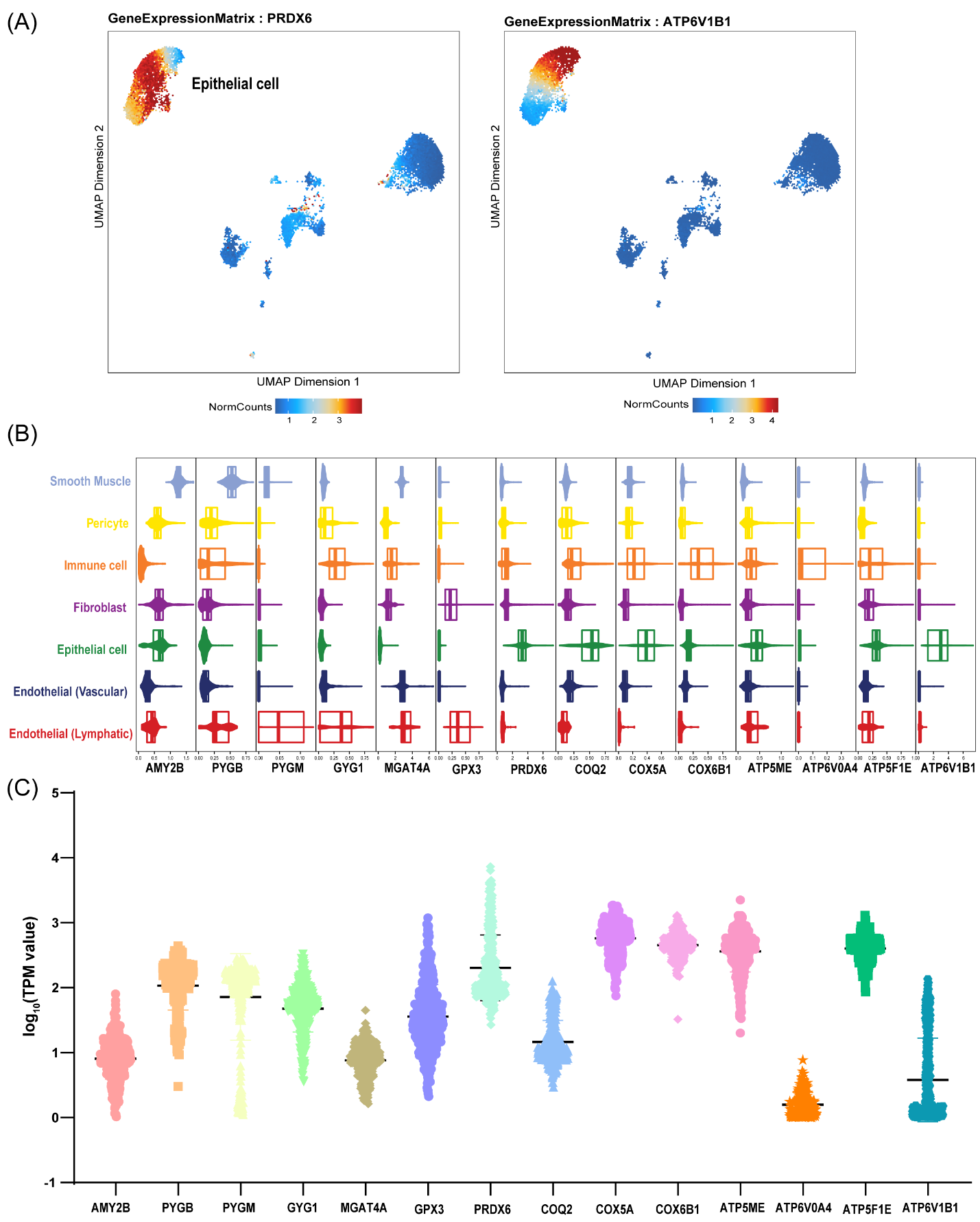


**Figure S12.** The expression of candidate genes in bovine rumen snRNA-seq and bulk RNA-seq data. (A) UMAP plot showing the rumen epithelial cell specific marker genes. (B) The expression of 14 genes in rumen different cell types. (C) The expression of 14 genes in rumen tissue. (**Unpublished snRNA-seq data**)
